# New approach methodologies in nanomedicine: bridging the gap between *in silico*, *in vitro*, and *in vivo* systems

**DOI:** 10.1039/d6cs01323f

**Published:** 2026-10-15

**Authors:** Yasmine Radwan, Laura P. Rebolledo, Elizabeth Skelly, Emma Grabarnik, Sankalp Jain, Morgan Halman, Roger Chammas, Alexey V. Zakharov, Marina A. Dobrovolskaia, Kirill A. Afonin

**Affiliations:** a Chemistry and Nanoscale Science Program, Department of Chemistry, University of North Carolina at Charlotte Charlotte NC 28223 USA kafonin@charlotte.edu; b MIMETAS US Gaithersburg MD 20878 USA; c Nanotechnology Characterization Laboratory, Cancer Research Technology Program, Frederick National Laboratory for Cancer Research Frederick MD 21701 USA marina@mail.nih.gov; d National Center for Advancing Translational Sciences, National Institutes of Health Rockville MD 20850 USA; e Center for Translational Research in Oncology (LIM/24), Instituto do Cancer do Estado de Sao Paulo, Hospital das Clinicas HCFMUSP, Faculdade de Medicina, Universidade de São Paulo São Paulo 01246-000 Brazil; f Comprehensive Center for Precision Oncology (C2PO), Universidade de São Paulo São Paulo 01246-000 Brazil

## Abstract

The rise of nanomedicine has led to the regulatory approval of more than 80 nanoparticle (NP)-based therapeutics over the past two decades. Further advancement in the field requires a deeper understanding of the nano-bio interface of NPs, including their uptake, delivery, efficacy, toxicity, and adverse effects. Despite the heavy reliance on conventional cell cultures for NP screening, these models do not recapitulate the *in vivo* microenvironment, complexity, and physiological responses; hence, follow-up animal studies are required. Nonetheless, because of species-specific differences and ethical concerns around animal studies, developing alternative platforms has become critical. To address these issues, 3D cell culture models such as organoids, spheroids, and organ-on-chip systems have emerged to bridge the gap between *in vitro* and *in vivo* systems. Because they better represent cell interactions, gene expression, nutrient gradients, and tissue-specific functions, 3D models also align with the U.S. FDA's promotion of new approach methodologies (NAMs) to reduce animal testing. This review covers examples of organic and inorganic NPs tested in 3D models and discusses organ-specific NAMs, considering their advantages and limitations. The review also highlights how 3D models improve NP evaluation for drug delivery, toxicity, and targeted therapy, and provides a coherent NAMs toolbox for developing *in silico* methods for NP assessment. Future perspectives are introduced to underscore the importance of integrating *in silico* NAMs and 3D models to complement *in vivo* models for advancing precision medicine and improving the translational potential of complex drug products containing nanotechnology.

## Abstract 2

Rapid advances in biomedical research and nanomedicine have driven the development and implementation of New Approach Methodologies (NAMs) for predictive evaluations of novel formulations.

## Graphical abstract

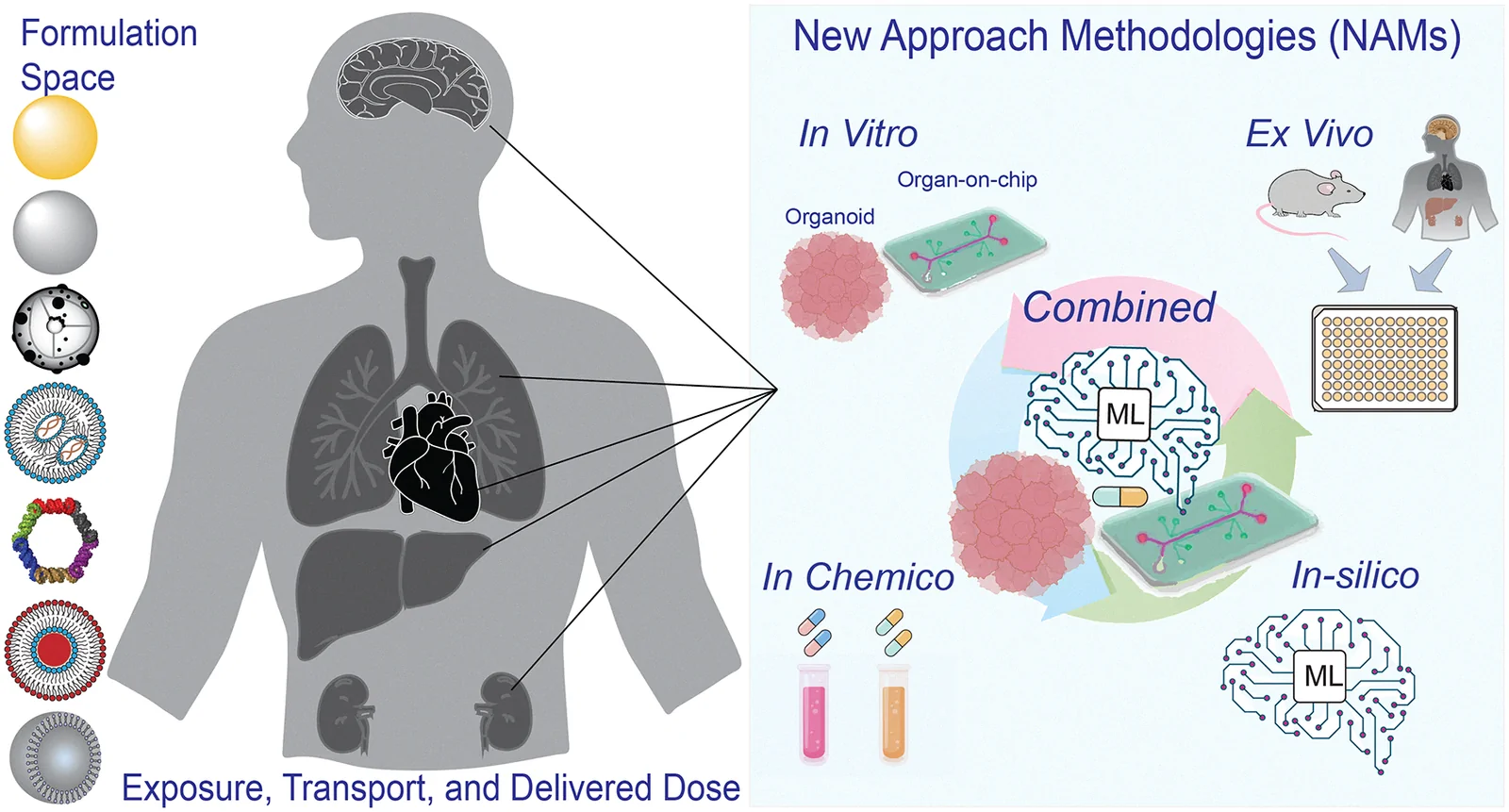

## 1. Introduction

Nanomedicine is the branch of medicine that uses nanotechnology to advance therapeutics and diagnostics.^1,2^ Nanoparticles (NPs) are discrete particulate structures with at least one external dimension measuring in the nanoscale range.^3^ However, there is no formal definition of the nanoscale limits for a medical device or a drug product to be considered as nanotechnology-containing. For example, according to the national nanotechnology initiative, NPs are objects with a size in at least one dimension between 1 and 100 nm; according to the US Food and Drug Administration (FDA), a drug product is considered nanomaterial if it has a size up to 1 µm.^4^ According to the FDA, the key factor distinguishing an engineered nanomaterial from natural nano-sized molecules is the size-related properties.^4^ For example, engineered nanomaterials have a very high surface-area-to-volume ratio and optical, magnetic, electronic, or catalytic properties that differ from those of the corresponding bulk material and vary with particle dimensions. NPs may be organic or inorganic in composition and can be tuned largely independently in size, shape, rigidity, surface charge, and surface chemistry, and it is this combination of adjustable parameters that allows them to be engineered for a specific task.^5–7^ The growing interest in NP-based therapeutics has led to the approval of around 80 nanomedicine-based products over the last 20 years by the FDA and the European Medicines Agency (EMA) for commercialization.^8^ The increasing interest in nanomedicines can be seen by looking at the FDA approval timeline (Fig. 1), which further emphasizes the contributions of nanomedicine to the diagnosis and treatment of diseases.

**Fig. 1:**
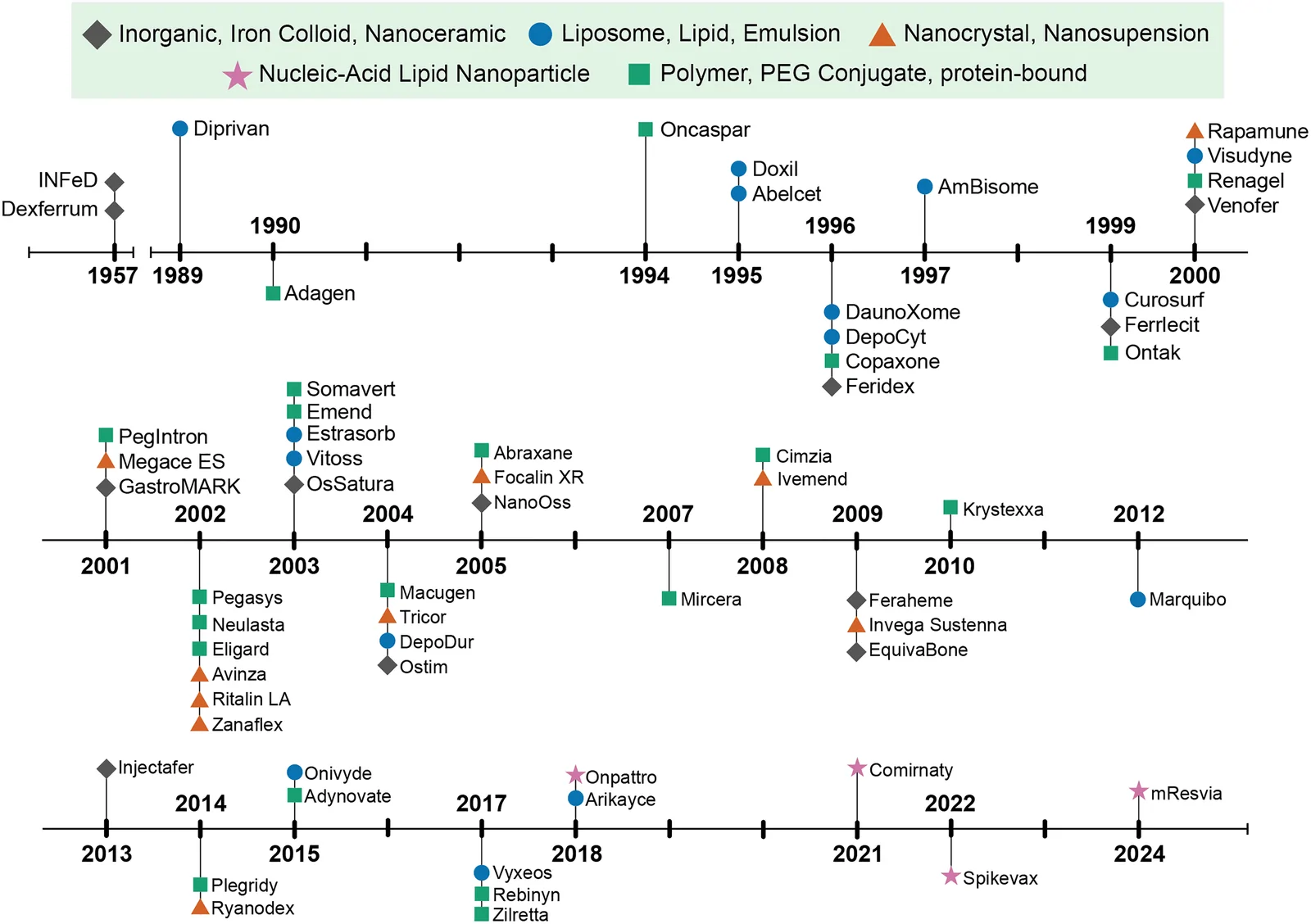
The timeline of nanomedicine approval by the FDA. The figure presents the timeline of approval of drug products containing nanotechnology from 1957 to 2024. The trademarks are used for information purposes only. The figure summarizes the nanotechnology platform used in each formulation at the top of the image.

To continue advancing the field, a deeper understanding of the nano-bio interface is needed. Because the efficacy and safety of new nanotechnology-based products must be thoroughly investigated,^9^ research continues to optimize existing models and analytical frameworks. Additionally, evolving legal, ethical, and regulatory landscapes provide additional feedback to shape scientific approaches. Moreover, technological advancements influence scientific approaches by allowing researchers to test previously inaccessible parameters. As such, the development, characterization, and validation of new and alternative models and methods have become an essential component of the research community's effort to optimize current approaches in drug discovery and development. Such optimization is an iterative process built on scientific knowledge, which in turn relies on constantly upgrading technologies pushing the limits of detection and opening opportunities to look deeper at molecular mechanisms at a single-cell or single-NP level while being relevant to the final user – a human patient. One example of such an iterative process is the evolution of cell-based models used in drug discovery and development.

Over the last few decades, assessment and high-throughput screening of NPs have heavily relied on *in vitro* assays in two-dimensional (2D) cell cultures. However, 2D cultures have many limitations, as they often fail to accurately depict *in vivo* conditions and microenvironments.^10^ This is because they poorly mimic *in vivo* biological functions and tissue organization, necessitating follow-up experiments in animal models, which are time- and resource-consuming.^10^ In addition, gene expression and protein regulation differ between animals and humans.^10^ Moreover, ethical concerns about using animals in experimental research add another layer of complexity and have led to the development of the 3Rs (replacement, reduction, and refinement) principle, which advocates using non-animal-based methods in research.^11^ As a result, three-dimensional (3D) cell culture technologies, including organoids, spheroids, organ-on-chip, and 3D bioprinting, have emerged over the last decade to bridge the gap between conventional cell cultures and *in vivo* systems.^12^ The use of 3D models is growing because they overcome the limitations of 2D cultures. For instance, in 2D cultures, cells grow in monolayers that lack interaction with the extracellular matrix (ECM); in 3D models, cells interact with and remodel the surrounding ECM, providing a more relevant representation of how tissues interact with the extracellular microenvironment in living organisms. This is attributed to the ability of cells in 3D cultures to grow and form cell–cell and cell–ECM interactions, thus allowing them to proliferate and differentiate while mimicking *in vivo* cell behavior and morphology.^11,13,14^ Another advantage of 3D models over 2D cultures is nutrient distribution and accessibility: 2D cultures have homogeneous nutrient distribution, whereas 3D models create nutrient gradients that mirror *in vivo* conditions.^11^ Hence, 3D models are more physiologically relevant, as they improve assessments of biological mechanisms, including viability, proliferation, differentiation, morphology, gene expression, drug metabolism, and response to stimuli.^14^ As a result, researchers increasingly use 3D models to study organ physiology and disease.^11^

The relevance and promising applications of 3D models are highlighted by the movement of the FDA and the National Institutes of Health (NIH) to increase the use of new approach methodologies (also called new approach methods and new alternative methods), hereby referred to as NAMs, to reduce animal testing in preclinical research and increase the relevance of preclinical data to humans.^15–19^

The U.S. FDA Center for Drug Evaluation and Research (CDER) considers a preclinical drug development model to be a NAM when it is “conducted *in vitro*, *ex vivo*, *in silico* (computational), or in chemico (chemical-based); improves the predictivity of clinical outcomes; shifts studies to phylogenetically lower animals; or otherwise helps replace, reduce, and refine animal use”.^20–22^Fig. 2 outlines the NAMs and their contribution to the 3R principle and personalized medicine.

**Fig. 2:**
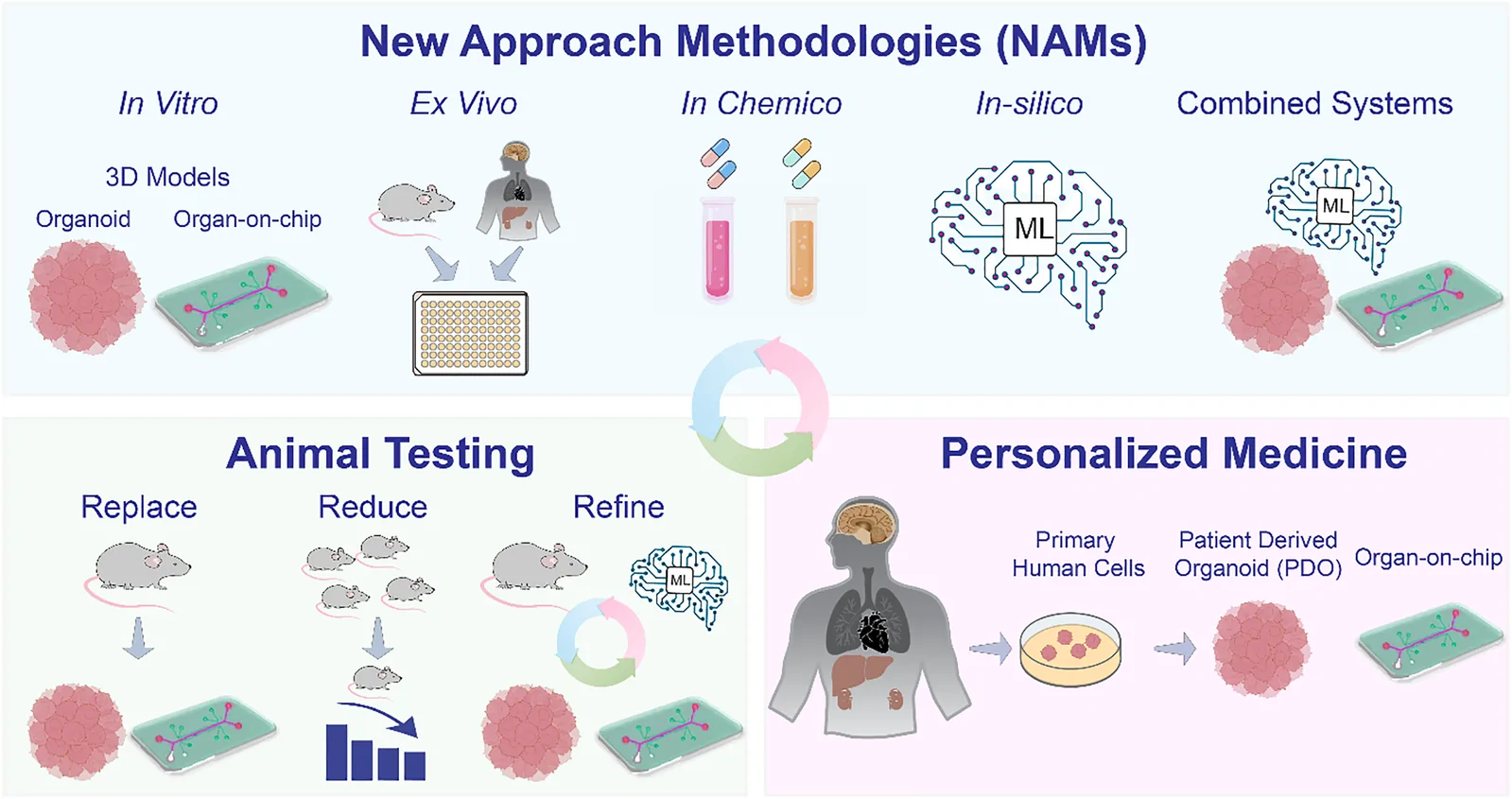
New approach methodologies (NAMs) and their role in improving clinical outcomes predictivity and contributing to the 3R (Replacement, Reduction and Refinement) principle. This figure summarizes different types of NAMs, how they relate to the animal testing 3R principle, and their use in personalized medicine.

Organoid models are a broad category of *in vitro* organ models derived from stem cells, referred to in the current literature as organoids, or tissues obtained from patients, referred to as patient-derived organoids or PDOs.^23^ Induced pluripotent stem cells (iPSCs), surgically removed tumor tissues, and peripheral blood-derived circulating tumor cells were also used to establish tumor organoids, also called cancer organoids or tumoroids; for a patient-derived source, the term patient-derived tumoroids (PTO) is used.^23^ Organoids and tumoroids vary in size, growth rates, and culture requirements, with the majority utilizing Matrigel as an extracellular matrix (though other types of matrices are also used) and a variety of growth factors, small molecules, and molecule inhibitors, which are specific to each type of tumoroid (reviewed in ref. 23).

Organ-on-chip platforms are another category of 3D *in vitro* NAMs that offer additional engineering control over the 3D architecture and multicellular interactions within microfluidic platforms, enabling controlled rates of fluid flow, shear stress, and mechanical strain. These devices can incorporate multiple cell types from healthy or diseased states, supporting the growth of perfusable microvasculature and the incorporation of molecular gradients and circulating immune cells.^24^ The ability to tailor these models to recapitulate key mechanistic events and cell-drug interactions related to adverse outcomes is a valuable feature, particularly for the development of new drug modalities.^25^

The further development of 3D cultures to model organ physiology and diseases has allowed researchers to model many organs and disease models, including the brain, kidney, liver, and cancer.^11^ These models are widely used in nanomedicine for applications such as NP-based drug delivery^26–30^ and targeted therapy,^31–33^ nanotoxicology and safety assessment,^9,34,35^ and imaging.^36,37^ However, to date, NAMs are not yet a common approach for testing nanomedicines, and scientific reviews often still expect animal studies,^38^ even in very early discovery stages. The modularity of NPs and their ability to independently tune size, shape, rigidity, surface charge, and surface chemistry has enabled the systematic generation of large NP libraries that are highly compatible with high-throughput screening.^39^ While testing these large libraries *in vivo* may be prohibitively expensive, and testing only a few compounds may be insufficient to draw conclusions, NAMs offer a more robust approach to investigate early safety and efficacy and define mechanistic structure–activity relationships, which are most pertinent.^40^ Nanomedicine is well positioned to leverage NAMs for fail-fast, high-throughput screening, which is currently underutilized.

This review discusses 3D models and the types of NPs used in them, highlighting organ-specific models (*e.g.*, brain, liver, kidney, and cancer) and considering their advantages and limitations in the broader context of NAMs. The review highlights how 3D models enhance the evaluation of NPs for drug delivery, toxicity, and targeted therapy. In addition, the review provides a coherent NAMs toolbox for *in silico* methods used for NPs assessment (Fig. 3). Future perspectives are discussed to underscore the importance of integrating *in silico* models with 3D models to advance personalized medicine and translational potential in the nanomedicine field. Table 1 lists applications of NPs using NAMs and highlights key findings.

**Fig. 3:**
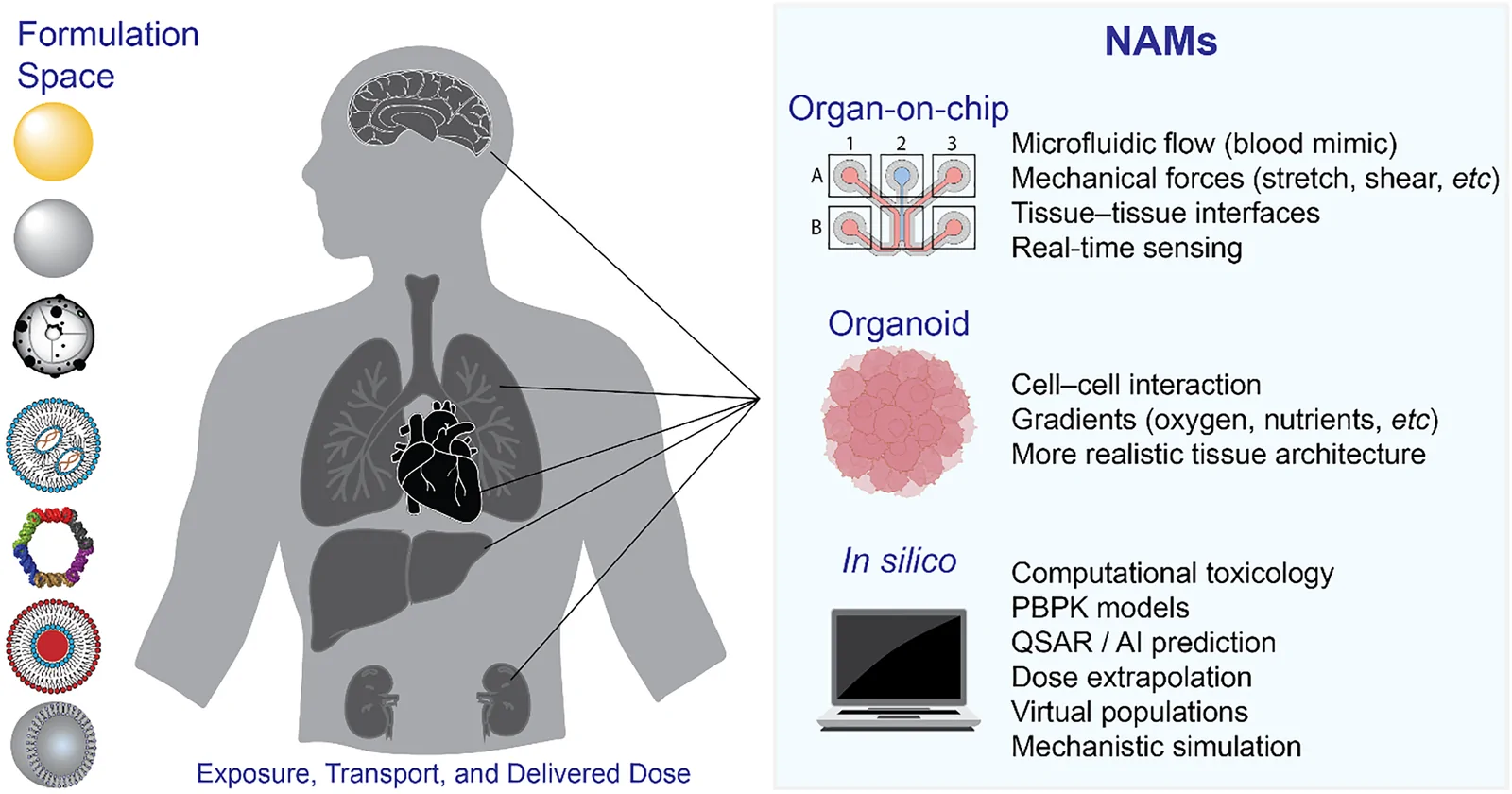
NP exposure and NAMs overview. NPs are used for drug delivery *via* various routes. The most popular route, and the one used for currently approved nanomedicines, is injection. However, researchers are developing NPs for oral, intranasal, intracranial, and other routes of administration to achieve tissue-specific drug delivery. The breadth of nanotechnology carriers, formulation technologies, and targeting strategies enables specific delivery of cargo to disease-affected cells and tissues. NAMs, including organ-on-chip, organoids, and *in silico* approaches, can help to study tissue interactions and better understand dose responses and toxicity of NP formulations in organs (*e.g.*, brain, liver, kidneys).

**Table 1:** Examples of applications of NPs utilizing NAMs

Application	Model system	Structural complexity	Key finding
Drug delivery	3D Glioblastoma organoid	GBM organoids were developed from six human brain cell types, providing an integral BBB.	Ultrasmall gold NPs (AuNPs) functionalized with doxorubicin and AF647 enhanced the drug permeability to the BBB and increased its efficacy.^41^
Targeted therapy	Human kidney and liver organoid-based multi-organ-on-a-chip	Kidney-injured tubuloids and liver organoids combined by microfluidic channels provide a model for acute renal injury.	Mesenchymal stromal cell (MSC)-derived small extracellular vesicles (SEVs) stimulated recovery of the renal epithelium after injury, with therapeutic efficacy that recapitulates animal models.^42^
Toxicology	3D Liver spheroids	Hepatocellular carcinoma model cultured from HepG2 cells.	Polymeric NPs induced a lower dose- dependent hepatotoxicity in 3D spheroids as they have a more *in vivo*-like phenotype than the 2D culture.^9^
Immunology	Artificial immune cell (AI-cell)	Transformer-based, computational, sequence-focused model designed to simulate immune system responses to nucleic acid NPs (NANPs).	NANP designs are guided toward targeted immune responses based on sequences.^43,44^
Imaging	Tumoroid	Artificial cancer mass (ACM), surrounded by uncompressed collagen populated by fibroblasts to mimic cancer stroma. The model mimics the tumor microenvironment.	AuNPs’ uptake distribution within the tumoroid imaged *via* XRF, measured a AuNPs challenging concentration ratio of 5 : 1 between the ACM and stroma of the tumoroid.^37^

## 2. Types of NPs reported in 3D systems

Organic NPs constitute a highly adaptable class of nanocarriers for drug and nucleic acid delivery, owing to their modular chemistry, biocompatibility, and functional chemistry^45^ (Fig. 4). In 3D culture models such as spheroids and organoids, organic NPs must navigate dense cell layers and extracellular matrices, leading to behaviors that better reflect *in vivo* conditions than those seen in 2D cultures. Accordingly, this section focuses on polymeric NPs, including nucleic acid NPs (NANPs), lipid-based NPs, and dendrimers, as representative organic systems investigated in 3D cultures.

**Fig. 4:**
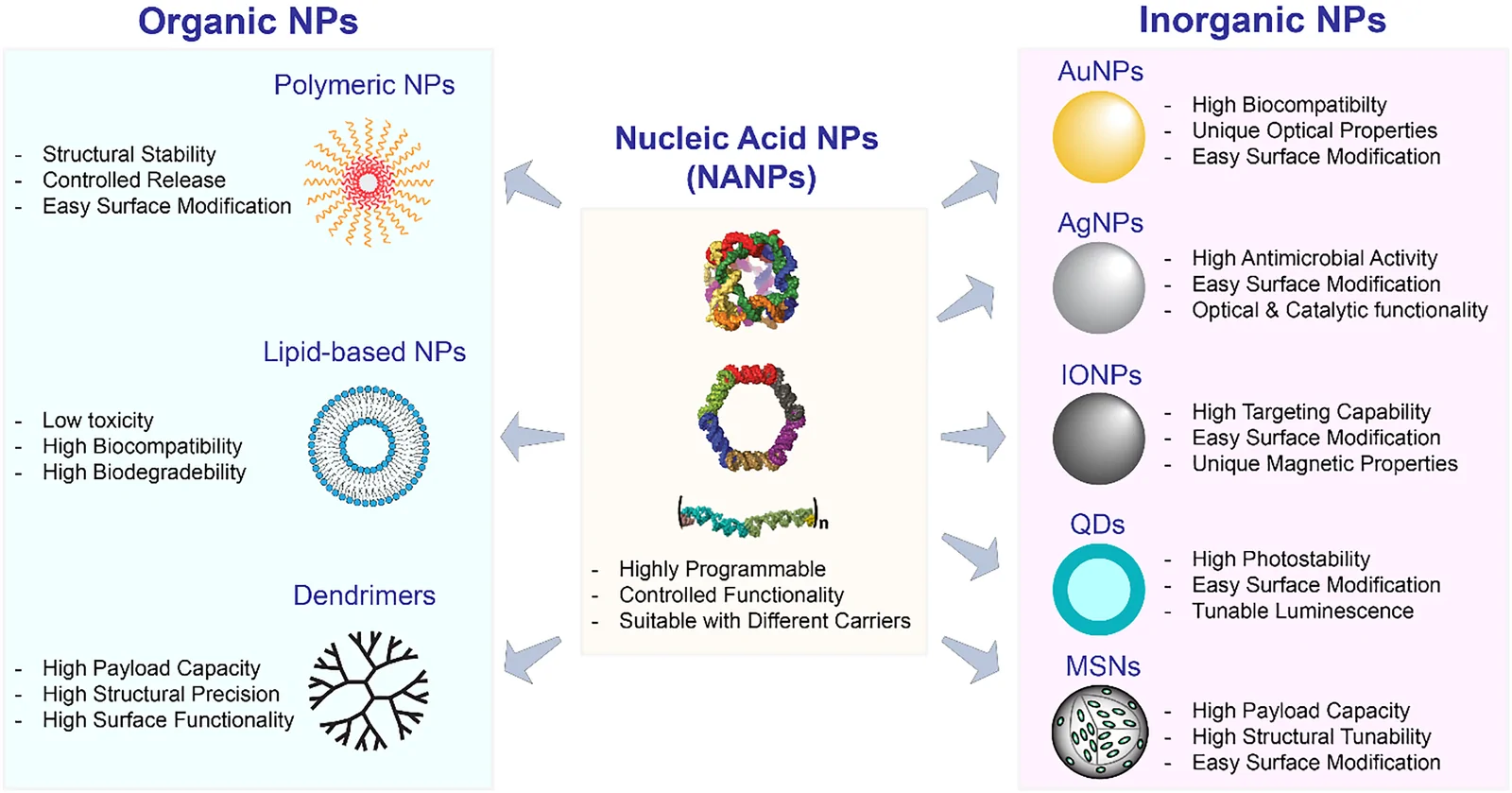
Schematic illustrating organic and inorganic NPs, highlighting their advantages. NANPs fall under the organic NPs category. However, because of their versatility and intended intracellular application, NANPs can be complexed with organic or inorganic NPs to serve as carriers for intracellular delivery and uptake.

Polymeric NPs are among the most widely explored organic nanocarriers in 3D culture systems because of their structural stability, controlled-release capabilities, and ease of surface modification. Common polymers include poly(lactic-*co*-glycolic acid) (PLGA), polyethyleneimine (PEI), chitosan, and polyethylene glycol (PEG)-based copolymers.^46^ In 3D tumor spheroids, polymeric NPs show diffusion limitations, accumulation gradients, and delayed uptake kinetics absent in 2D monolayers, highlighting the importance of 3D models for evaluating NP penetration and therapeutic efficacy.^47,48^ The characteristic properties of these polymers, the advantages and limitations that follow from them, and the circumstances in which each is preferred are summarized in Table 2.

**Table 2:** Polymers commonly used to formulate NPs evaluated in 3D culture systems. The table summarizes polymers’ characteristic properties, the advantages and limitations that follow from those properties, and the circumstances in which each is the preferred choice

Polymer	Key properties	Principal advantages	Main limitations	When it is preferred
PLGA	Linear copolymer of lactic and glycolic acid; hydrolytic degradation to endogenous monomers; degradation rate tuned from weeks to months by the lactide:glycolide ratio and molecular weight; hydrophobic core, near-neutral to slightly anionic surface.^49^	Clinically approved with a well-characterized safety record; sustained and tunable release; efficient encapsulation of hydrophobic small molecules; degradation products cleared through normal metabolism.	Poor loading of hydrophilic or anionic cargo such as nucleic acids; acidification of the particle interior during degradation can destabilize sensitive payloads; initial burst release; little intrinsic endosomal escape.	Sustained delivery of hydrophobic small-molecule drugs, and studies in which prolonged exposure of a spheroid or tumoroid to a slowly releasing depot is the desired regimen.^49^
PEI	Branched or linear polycation of high charge density with extensive secondary and tertiary amine content, giving strong buffering capacity between pH 5 and 7; non-degradable; 25 kDa branched PEI is the field benchmark.^50^	Condenses nucleic acids efficiently into compact polyplexes; promotes endosomal escape through the proton-sponge effect; among the highest transfection efficiencies of any non-viral carrier.	Cytotoxicity that increases with molecular weight and charge density; no biodegradation, so repeated dosing accumulates polymer; aggregation and protein-corona formation in serum-containing media.	Nucleic acid delivery where transfection efficiency outweighs tolerability, and as the standard positive-control comparator; in 3D constructs it is also informative for probing how cationic charge drives binding to the outer cell layers.^47,50,51^
Chitosan	Cationic linear polysaccharide obtained by deacetylation of chitin; charge is pH dependent with a pKa near 6.5; mucoadhesive; degraded enzymatically by lysozyme.^46^	Biodegradable, inexpensive and well tolerated; mucoadhesion prolongs residence at epithelial surfaces; transiently opens tight junctions to enhance paracellular transport; complexes nucleic acids without organic solvents.	Limited solubility above pH 6.5, which reduces colloidal stability at physiological pH; substantial batch-to-batch variation in molecular weight and degree of deacetylation; transfection efficiency lower than PEI.	Mucosal and epithelial applications such as oral, nasal and pulmonary delivery, and any 3D model in which barrier opening or mucoadhesion is the property under investigation.^52^
PEG-based copolymers	Amphiphilic block copolymers in which a hydrophilic, electrically neutral PEG corona surrounds a hydrophobic core such as PLGA or PLA; the corona binds a hydration shell that sterically shields the particle surface.^47,53^	Reduces opsonization and phagocytic clearance, extending circulation time; improves colloidal stability and suppresses aggregation; the neutral hydrated corona limits adhesive trapping in extracellular matrix and mucus, which improves diffusion into tissue.	The same shielding that aids transport reduces cellular uptake and endosomal escape, the so-called PEG dilemma; anti-PEG antibodies can cause accelerated clearance on repeated administration.	Situations requiring deep penetration through dense matrix or mucus, or prolonged circulation; PEGylation is the standard first strategy when a formulation fails to reach the core of a spheroid or organoid.^47,53^

NANPs are a distinct, highly programmable subclass of polymeric NPs assembled exclusively from RNA, DNA, or their chemical analogs into defined architectures.^54–57^ NANPs enable precise control over size, shape, and functionality, allowing the simultaneous delivery of therapeutic nucleic acids and immunomodulatory elements.^54^ In addition, NANPs can serve as active pharmaceutical ingredients (APIs), excipients, or both, depending on the intended therapeutic application.^58^ However, for intracellular delivery, NANPs must be complexed with a carrier. Multiple nanocarriers have been used for NANP delivery, including mesoporous silica NPs (MSNs),^58,59^ iron oxide NPs (IONPs),^60^ polymeric NPs,^61^ quantum dots (QDs),^62^ and lipid NPs (LNPs).^63,64^ Carrier type and NANP composition influence internalization, subcellular localization, and immunostimulation.^58,63,65^ In 3D culture systems, NANPs have shown structure-dependent cellular uptake, immune activation, and spatial distribution within multicellular constructs.^66–68^ Importantly, 3D models provide a physiologically relevant platform to assess NANPs’ stability, intracellular trafficking, and immunological responses, parameters that are often misrepresented in 2D cultures.^66–68^

Lipid-based NPs, including liposomes and LNPs, are clinically validated carriers for nucleic acids and small-molecule therapeutics.^69^ Their amphiphilic nature facilitates membrane fusion and endosomal escape, making them particularly effective for intracellular delivery.^69,70^ In 3D culture models, lipid-based NPs encounter ECM networks and heterogeneous cell populations that significantly influence their penetration depth and biodistribution.^64,71^ Studies using tumor spheroids and organoids have demonstrated that lipid-based NP performance in 3D systems more accurately predicts *in vivo* delivery outcomes, including reduced penetration into hypoxic or necrotic cores and altered transfection efficiencies. Consequently, researchers increasingly use 3D models to optimize lipid composition, particle size, and surface charge before *in vivo* studies.

Dendrimers are highly branched, monodisperse macromolecules with defined architecture and high surface functionality. Poly(amidoamine) (PAMAM) dendrimers are among the most extensively studied in 3D culture systems for drug and gene delivery applications.^66,72–75^ Their multivalent surface allows for high payload loading and targeted ligand attachment. However, their transport, penetration, and cellular interactions are strongly influenced by ECM density, spatial constraints, and diffusion barriers that are not recapitulated in 2D monolayers. Consequently, 3D culture models are critical for accurately evaluating dendrimer distribution, penetration depth, and therapeutic efficacy within physiologically relevant tissue-like environments.

Collectively, organic NPs behave differently in 3D culture models than in 2D systems, underscoring the need to incorporate 3D platforms into NP design and optimization. By capturing key physiological barriers such as ECM interactions, cell–cell communication, and diffusion constraints, 3D culture models provide a critical bridge between *in vitro* screening and *in vivo* translation for organic NP-based therapeutics.

Inorganic NPs composed of gold (AuNPs), silver (AgNPs), iron oxide (IONPs), silica (SiO_2_-NPs), and QDs have been shown to accumulate in various organs (heart, lungs, kidneys, *etc.*) based on material, size, charge, and surface modifications, with low clearance and high toxicity.^76–78^ These undesired side effects have led to smarter design strategies that enable faster clearance and reduced toxicity; higher clearance rates can depend on NP size (less than 10 nm) and the introduction of inorganic or organic surface modifications^78^ (Fig. 4). Unfortunately, the clearance and toxicity of NPs are difficult to predict in 2D cell culture, as it does not capture the complexity of biological models.^79^ Recent studies have evaluated the therapeutic abilities and toxicity of inorganic NPs using 3D cell culture to better mimic the complexity of *in vivo* studies.^79^

AuNPs have been synthesized and evaluated in various shapes, including nanorods and nanospheres, with sizes ranging from less than 2 nm to 100 nm.^80^ In addition, their surfaces can be functionalized with either inorganic or organic ligands, depending on the intended payload application.^81^ AuNPs are commonly studied because they can aid photodynamic therapy and serve as imaging agents.^81^ At less than 2 nm, ultrasmall AuNPs can pass through the blood–brain barrier, as supported by 3D cell culture work.^41^ When combined with therapeutics and used in photodynamic therapy, these ultrasmall AuNPs have been shown to reduce the size of 3D tumor spheroids.^82^

AgNPs are multifunctional in that they kill bacterial, fungal, and mammalian cells through multiple mechanisms, such as the production of reactive oxygen species (ROS), endoplasmic reticulum stress, disruption of protein folding, destruction of nucleic acids, and mitochondrial damage.^83^ AgNPs have been studied and used primarily for their inherent antibacterial properties without high toxicity to mammalian cell cultures. This apparent selectivity warrants caution. The mechanisms responsible for bacterial killing, namely ROS generation, oxidation of proteins and nucleic acids, and loss of membrane integrity, are not specific to prokaryotes, and the same chemistry acts on host cells; the therapeutic window reflects differences in sensitivity and in the dose reaching each compartment rather than any difference in mechanism. Mammalian cells are partially protected by glutathione and enzymatic antioxidant defenses and by a larger cytoplasmic volume, but these defenses are finite and can be exceeded at concentrations only modestly above antibacterial ones, particularly in metabolically active cells or in tissues where particles accumulate. Any antimicrobial application therefore requires the host-cell dose–response to be established alongside the antibacterial one.^84,85^ However, toxicity depends heavily on the synthesis method, surface modification, capping agent, and reducing agent used during synthesis.^83^ Therefore, it is important to evaluate AgNPs in a variety of mammalian cell systems before systemic use. AgNPs have been evaluated in 3D cell culture systems, including fibroblast and melanoma spheroids, where AgNPs were found to be more cytotoxic in 3D cell culture than in 2D cell culture.^86^ In some models, such as the 3D EpiKutis model mimicking human skin, AgNPs were not found to distribute within the 3D cell culture, whereas the NPs were evenly distributed in 2D cell culture keratinocytes, highlighting the limitations of these particles and the differences between 2D and 3D cell culture.^87^ Finally, high-Z metals, such as Au and Ag, have also been tested as radiosensitizers with promising results accumulating in the literature for more than a decade.^88–90^ Translation of the *in vitro* and *in vivo* findings has been slowed by the paucity of adequate models to assess their potential toxicities and overall safety, a gap to be filled with the development of NAMs.^91^

IONPs have been used in multiple biomedical applications, including magnetic resonance imaging (MRI) as contrast agents, targeted drug delivery, cell tracking/biosensing, and antibiotic applications.^92^ Unfortunately, broader biomedical applications are limited by cell toxicity and IONP oxidation; however, like AgNPs, these limitations depend on size, shape, and particle coating. The coating on IONPs ranges from polymers to organic surfactants or inorganic compounds to bioactive molecules, which can be functionalized further with drugs.^92^ Initially, IONPs were found to be biocompatible and nontoxic to mammalian cells;^93^ however, when moving to *in vivo* models, IONPs were found to accumulate in various tissues and cause DNA damage, oxidative stress through the production of ROS, mitochondrial dysfunction, and cell membrane damage leading to cellular death.^94^ This highlights the need for more complex cell models, such as 3D cell culture, that better mimic *in vivo* models.

Core–shell QDs are typically composed of either II to VI or III to V elements with tunable luminescence characteristics, including a wide excitation and narrow emission spectra, depending on the size of the particle and bandgap of the material.^95^ Cadmium selenide (CdSe) and zinc sulfide (ZnS) QDs are commercially available with surface modifications for use in targeted cell tracking, bioimaging, and biomarker detection. The wide range of QD sizes (2.5–100 nm), compositions (*e.g.*, Cd, Se, Pb), and capping agents (*e.g.*, mercaptopropionic acid, mercaptoundecanoic acid, cysteamine) makes it difficult to broadly classify them as biocompatible or cytotoxic. However, there are examples of *in vitro* studies that show QDs to be toxic to mammalian cells, causing cell death through QD core degradation and capping agent.^96,97^ The toxicity of the CdSe core is mechanistically well defined and arises mainly from Cd^2+^ release following oxidative or photolytic core degradation. Free Cd^2+^ binds sulfhydryl groups on mitochondrial proteins, impairing respiration and generating further ROS, and displaces zinc from zinc-finger transcription factors and DNA repair enzymes, which accounts for genotoxicity at concentrations below those causing overt cytotoxicity. Cadmium is additionally a recognized human carcinogen with a biological half-life in the kidney measured in decades, so slowly cleared particles constitute a reservoir of prolonged exposure rather than an inert label. A ZnS shell suppresses but does not abolish this release, because shell integrity is compromised by oxidation, ultraviolet illumination, and the acidic environment of the endolysosomal compartment, which are precisely the conditions encountered after cellular uptake. Apparent biocompatibility measured over the 24- to 48-hour windows typical of 2D assays may therefore understate the hazard, and the longer culture periods sustainable in 3D systems are better suited to capturing degradation-dependent toxicity.^98,99^ Alternatively, several studies have demonstrated a lack of, or concentration-dependent, cytotoxicity.^100,101^ Like other inorganic NPs, 2D does not show the full cytotoxicity that QDs have on 3D cell culture.^102,103^ As QDs become more commercially available for biomedical and bioimaging applications, it is important to understand each QD type separately, and it is necessary to test these particles in 3D cell culture to better assess their cytotoxicity.

MSNs have emerged as versatile nanocarriers for drug and nucleic acid delivery due to their high surface area, tunable pore size, and ease of surface functionalization.^104^ Before loading, the mesopores of a typical MCM-41-type MSN are ordered cylindrical channels of roughly 2 to 3 nm in diameter, expandable to about 10 nm or more using swelling agents or alternative templating, which gives specific surface areas in the region of 700 to 1000 m^2^ g^−1^ and pore volumes approaching 1 cm^3^ g^−1^.^105,106^ These dimensions determine what can be accommodated: small-molecule drugs load readily into unmodified pores, whereas siRNAs, approximately 2.5 nm across and 7 to 8 nm long, require enlarged pores or electrostatic adsorption to the external surface, and most proteins need pore diameters above 10 nm.^107,108^ While their performance has been extensively characterized in 2D monolayer cultures,^109,110^ 3D model systems, such as multicellular spheroids and organoids, provide a more physiologically relevant platform to evaluate MSN transport, penetration, and therapeutic efficacy.^111^ In 3D systems, MSNs must overcome additional biological barriers, including dense extracellular matrix deposition, limited diffusion gradients, and hypoxic cores, which more closely mimic *in vivo* tumor microenvironments. Studies using 3D spheroids have demonstrated that MSNs’ size, surface charge, and functional coatings (*e.g.*, PEGylation or targeting ligands) critically influence their penetration depth and cellular uptake.^53,112^ Importantly, 3D models enable assessment of controlled cargo release kinetics and therapeutic response under conditions that better reflect *in vivo* drug resistance and cellular heterogeneity. Beyond conventional chemotherapeutic and nucleic acid delivery applications, MSNs have also been explored as carriers for photosensitizers (*e.g.*, chlorin e6) in photodynamic therapy (PDT),^113^ where their high surface area and tunable pore structure enhance photosensitizer loading, solubility, and tumor selectivity. Such approaches have shown promise in difficult-to-treat malignancies, including pancreatic cancer and triple-negative breast cancer.^113–116^ Therefore, integrating MSNs into 3D culture platforms provides a more predictive framework for optimizing NP design prior to *in vivo* evaluation.

## 3. Methods for NP characterization

Interpreting any 3D experiment depends on knowing what was applied to the cells, and the properties that govern NP behavior in a biological environment are neither fixed nor captured by a single measurement. Characterization is therefore best treated as a panel of orthogonal measurements performed on both the as-synthesized material and the dispersion as delivered, since size, charge, and aggregation state can all change upon transfer into cell culture medium. The measurands that matter most for 3D work are NP size and size distribution, morphology, surface charge, surface chemistry and ligand density, porosity where relevant, composition and purity, colloidal stability in the exposure medium, and the identity of the adsorbed protein corona.^117^

Researchers commonly measure size by dynamic light scattering (DLS), which reports an intensity-weighted hydrodynamic radius and a polydispersity index. Because scattering intensity scales with the sixth power of NP radius, DLS is disproportionately sensitive to a small number of aggregates and should not be relied on alone. Nanoparticle tracking analysis (NTA) and tunable resistive pulse sensing provide number-weighted distributions and NP concentrations, while transmission electron microscopy (TEM) directly provides core dimensions and morphology. Cryogenic TEM (cryo-EM) is preferable for lipid and polymeric systems that are distorted by drying, and small-angle X-ray scattering (SAXS) resolves internal organization, such as lamellarity or nucleic acid packing within an LNP. Surface charge is inferred from electrophoretic mobility and expressed as a zeta potential, a quantity strongly dependent on the ionic strength and pH of the suspending medium; values obtained in water do not transfer to complete culture medium, and the measurement medium should always be reported alongside the value.^118^

For porous carriers such as MSNs, nitrogen physisorption yields the specific surface area by the Brunauer–Emmett–Teller method and the pore size distribution by density functional theory or Barrett–Joyner–Halenda analysis, which together define how much cargo a particle can accommodate and which cargoes will fit at all.^119^ Inductively coupled plasma mass spectrometry quantifies elemental composition and particle number concentration; its single-particle mode can size and count particles in biological matrices where optical methods fail. Surface composition is accessible by X-ray photoelectron spectroscopy (XPS), functional group identity by infrared (IR) and nuclear magnetic resonance (NMR) spectroscopy, crystallinity by X-ray diffraction (XRD), and the mass fraction of grafted organic material, and hence ligand density, by thermogravimetric analysis. Optical characterization by ultraviolet-visible (UV-vis) absorbance additionally reports the plasmon band position for gold and silver particles, while fluorescence spectroscopy with quantum yield determination is required for quantum dots.^120^

Properties measured in water rarely persist in biological settings. Upon contact with serum-containing medium, NPs acquire a protein corona that alters their effective size, charge, and the receptors they engage. Therefore, size and charge measurements should be repeated in the actual exposure medium, and the hard corona should be identified proteomically when the biological outcome depends on it.^121^ Colloidal stability over the duration of the experiment matters more in 3D than in 2D, because exposures typically run for days rather than hours. Endotoxin contamination must also be excluded using the limulus amoebocyte lysate (LAL) assay or a recombinant equivalent, since trace lipopolysaccharide produces cytokine responses indistinguishable from NP-mediated immunostimulation, a particular concern for the immunological endpoints described in this review.^122^

Characterization should ultimately focus on the dose that reaches the cells rather than the concentration added to the well. Sedimentation and diffusion determine how much material reaches a monolayer, and in a spheroid or organoid, geometry imposes an additional penetration gradient, so nominal and delivered doses can differ substantially in a size- and density-dependent manner.^123,124^ Computational dosimetry models, discussed further in the section on computational NAMs, allow estimating the delivered dose from measured particle size, density, and medium viscosity. Reporting delivered dose alongside nominal concentration, together with the medium in which each property was measured, makes results comparable across laboratories and between 2D and 3D formats.^125,126^

## 4. Mechanisms of NP cellular entry and barrier crossing

NPs do not cross the plasma membrane by passive diffusion as small lipophilic drugs do. With few exceptions, NPs are internalized *via* energy-dependent endocytic processes, and the route taken determines both their intracellular destination and the fraction of the dose that remains biologically available.^127^

Phagocytosis is restricted to professional phagocytes such as macrophages, Kupffer cells, and microglia, and is generally supported by opsonins, serum proteins that enhance uptake *via* phagocytic receptors. The remaining routes are collectively termed pinocytosis. Clathrin-mediated endocytosis accommodates NPs up to approximately 200 nm, is receptor-driven, and delivers cargo to early endosomes that mature and acidify toward lysosomes. Caveolae-mediated uptake handles smaller NPs of roughly 50 to 80 nm and can bypass the lysosomal route, which is advantageous for cargoes degraded at low pH. Macropinocytosis is a non-selective, actin-driven engulfment of large volumes of extracellular fluid, together with anything suspended in it, and accommodates objects up to several micrometers. Particle size, shape, rigidity, and surface charge determine which route predominates, and because it is the protein corona rather than the bare surface that the cell encounters, corona composition ultimately selects the receptors engaged.^128,129^

Two qualifications are important when interpreting 3D data. First, ultrasmall NPs below approximately 3 nm behave differently from conventional NPs and can traverse membranes *via* non-endocytic routes, reaching compartments, including the nucleus, that larger particles cannot. Second, uptake is not the same as delivery: material that reaches the endolysosomal compartment is degraded or exocytosed unless it escapes into the cytosol, and endosomal escape remains the principal bottleneck for nucleic acid cargoes.^130,131^

Crossing a cellular barrier imposes further requirements. At the brain endothelium, the paracellular route is closed by continuous tight junctions, so transport must be transcellular, either by receptor-mediated transcytosis using endogenous systems such as the transferrin or low-density lipoprotein receptors, or by adsorptive-mediated transcytosis driven by cationic surface charge; efflux transporters including *P*-glycoprotein and BCRP (breast cancer resistance protein) then return a substantial fraction of small molecules that do enter.^132^ In 3D culture, these mechanisms operate in series with diffusion between cells, which act as both barrier and sink, taking up particles and thereby reducing the dose available to the core. Distinguishing genuine barrier crossing from sequential uptake and release is therefore essential when interpreting penetration data obtained in 3D models.

## 5. Organ-specific highlights

### 5.1. Brain models

Some of the greatest challenges hindering the study and development of novel therapies for the central nervous system (CNS) and the brain are the complexity of the CNS and limited access to the brain due to the blood–brain barrier (BBB). Therapies for CNS disorders have been facing higher failure rates in clinical trials due to low accumulation of the drug in the brain, leading to unsatisfactory efficacy.^11,133^ Another challenge is precision delivery, which requires targeting specific brain regions to avoid adverse CNS effects.^133^ The BBB restricts entry to a small subset of circulating compounds, principally those of low molecular weight and high lipid solubility, whereas larger and more polar molecules are effectively excluded. The mechanism underlying this selectivity is not settled: textbook accounts attribute it to passive diffusion across the endothelial membranes, but it has been argued that transport through phospholipid bilayers is negligible in real biomembranes, which are predominantly protein rather than lipid, and that cellular uptake is instead mediated almost entirely by solute carrier proteins.^134^ That position remains contested, and others maintain that passive and carrier-mediated processes operate together.^135^ What is not in dispute is that the paracellular route is closed by continuous tight junctions and that efflux transporters such as *P*-glycoprotein and BCRP return many compounds to the blood, so the permeability of a given molecule reflects the net balance of influx, efflux, and transporter expression rather than lipid solubility alone. Hence, delivering and releasing therapeutics into the brain is challenging. Nanomedicine has played a critical role in developing organic and inorganic NPs that cross the BBB and help overcome limited access to the brain.^8^ However, to further characterize and assess the efficacy of these NPs, 2D cell cultures fall short because they cannot mimic the complex interactions between CNS cells and the microenvironment, as they fail to replicate synapses among neurons due to the single plane provided by the flask of 2D cultures. This notion highlighted the need for a robust model to assess the mechanisms underlying CNS disorders. Hence, researchers developed several 3D models to better represent human neuron features, including ECM^136^ and neuron–glia and neuron–neuron interactions that mimic *in vivo* synapses.^11,137,138^ Added to this, 3D brain organoids developed from iPSC-derived human microglia-like cells have been developed to mirror the transcriptome profile and functional properties of human microglia.^11,139^ Different brain organoids developed from human iPSCs were used to study various CNS disorders, ranging from neurodegenerative diseases such as Alzheimer's disease and Parkinson's disease to neurodevelopmental diseases including autism spectrum disorders.^11,140^ In addition, researchers introduced a BBB-on-a-chip to model BBB dysfunction and immune cell migration.^141^ In light of these developments and more, 3D models offer a strong opportunity to better understand brain physiology and the mechanisms underlying CNS disorders. Various studies have established that AgNPs induce neurotoxicity; however, these results are based on 2D cell culture models^142,143^ or animal models,^144,145^ both of which fail to recapitulate the complexity of human brain development, hindering relevant clinical translation. A recent study used hiPSC-derived cerebral organoids to assess AgNP-induced developmental neurotoxicity. The group measured the changes in gene expression post-exposure to AgNPs *via* RNA sequencing, and immunohistochemical analysis was applied to evaluate neural proliferation, differentiation, and apoptosis in cerebral organoids after treatment with AgNPs. The results show that AgNP exposure disrupted cerebral-organoid growth and that apoptosis induction is concentration-dependent. This study provides a new platform for assessing the neurotoxicity of nanomaterials and understanding the molecular and cellular mechanisms linked to it, bridging the gap between 2D cell culture models and animal models.^146^ Another study investigated AuNP BBB crossing. The group developed a multicellular 3D brain spheroid model composed of six cell types: neurons, astrocytes, microglia cells, pericytes, endothelial cells, and oligodendrocytes, that represent reproducible BBB functions and properties. Understanding the limitations of this construct is important. The spheroid is formed by self-assembly rather than by engineering a vessel: the parenchymal cell types aggregate first, and endothelial cells and pericytes added subsequently migrate to the surface, where they form an outer layer expressing the tight junction proteins ZO-1 and claudin-5 together with adherent junction proteins. Coverage of that surface is incomplete unless a staged assembly protocol is used, and even then, the result is a cell-dense outer shell rather than a polarized endothelial monolayer lining a perfused lumen.^147,148^ The construct therefore lacks the separate luminal and abluminal compartments, the shear stress, and the continuous vessel geometry of the neurovascular unit *in vivo*,^149^ so no true permeability coefficient can be derived, and compounds reach the interior only by traversing the outer cell layers. The model provides a reproducible multicellular barrier containing all relevant brain cell types, within which the relative penetration of different agents can be compared; in this restricted sense, the term BBB should be understood.

Ultrasmall gold NPs were surface functionalized with fluorescein, and their crossing through the BBB was investigated *via* confocal laser scanning microscopy, as a function of time. The results showed that the spheroid rapidly took up the fluorescent ultrasmall gold NPs, while the free dissolved fluorescent dye used as a control was mostly rejected. They also showed that the concentration of NPs inside the spheroid increased with incubation time. In addition, AuNPs diffused into the center of the spheroid rapidly after crossing the BBB. Viability studies confirmed that neither the AuNPs nor the dye is cytotoxic. Furthermore, the group studied the effect of hypoxia on the BBB and demonstrated enhanced uptake of fluorescent AuNPs and dissolved fluorescent dye, indicating that the BBB is more permeable and compromised, consistent with clinical knowledge that ischemic stroke causes BBB damage. Specifically, the oxygen deprivation used in this model reproduces BBB disruption, in which hypoxia loosens the junctional seal through downregulation and redistribution of claudin-5 and ZO-1; similar hypoxia-driven junctional disruption occurs during ischemic stroke,^150^ in chronic cerebral hypoperfusion, and in vascular dementia.^151^ In conclusion, this study highlights the potential of AuNPs to deliver functional therapeutics across the BBB.^152^ In summary, 3D brain models provide a promising system for studying CNS disorders and bridge the gap between 2D cultures and animal models. However, further development is needed to develop 3D brain models with increased multicellular complexity.

### 5.2. Liver models

To increase the successful clinical translation of NPs, their safety and ADME (absorption, distribution, metabolism, and excretion) profiles should be studied. The human liver is a complex and highly vascularized organ that plays a key role in determining these profiles in the human body, as it serves as the detoxification organ, sequestering and removing endogenous and exogenous materials from the body.^153,154^ Hence, non-specific liver accumulation and unintended hepatotoxicity should be investigated.

The liver is not a uniform mass of hepatocytes, but a spatially organized tissue, and 3D systems must reconstruct this organization. Hepatocytes are arranged in repeating hexagonal lobules through which blood flows from the portal triads at the periphery towards the central vein, and this flow establishes gradients of oxygen, nutrients and hormones that, together with morphogenetic signals, create a highly variable microenvironment in which key hepatic functions are expressed non-uniformly, a phenomenon termed zonation; single-cell transcriptomic maps indicate that approximately half of hepatocyte genes are expressed in a zonated manner.^155^ Periportal hepatocytes are specialized for oxidative metabolism, gluconeogenesis, and urea synthesis, whereas pericentral hepatocytes carry the highest cytochrome P450 activity and are consequently the region in which many xenobiotics are bioactivated and where toxicant-induced necrosis characteristically begins.^155^ Hepatocytes account for roughly 70–80% of the total liver cells. The remaining non-parenchymal population contains fenestrated liver sinusoidal endothelial cells lining the sinusoids, hepatic stellate cells residing in the space of Disse, Kupffer cells acting as resident macrophages within the sinusoidal lumen, and cholangiocytes lining the bile ducts.^156^ This architecture also governs how NPs are handled, since circulating particles must first escape capture by Kupffer cells, which perform most phagocytic activity in the liver, and only those smaller than the sinusoidal fenestrations can pass into the space of Disse and reach hepatocytes directly.^156^ The dose that any individual hepatocyte receives, and the enzymatic machinery available to act upon it, therefore both depend on position within the lobule. Reproducing this heterogeneity, meaning several cell types held in their correct spatial relationships together with the gradients that arise from directional flow, is the central challenge for *in vitro* liver models.

2D cell culture models fall short in providing more predictive and reliable results because they do not accurately mimic liver proliferation and morphology. To improve the realism of *in vitro* liver models, researchers have developed next-generation 3D models to more accurately evaluate hepatotoxicity and ADME profiles of nanomaterials.^154,157^ 3D liver models must remain functionally stable over time to support exposure studies, while also recapitulating the human liver's physiological environment by mimicking active metabolic functions and complex organ structure. Exposure of the liver to NPs can disrupt mitochondria, leading to imbalanced oxidative homeostasis and reduced liver function, cell viability, and increased DNA damage.^154,158^ Therefore, 3D liver models can offer a more accurate understanding of the mechanisms that cause liver injury under realistic exposure conditions. A study investigated NP-induced hepatotoxicity by developing a 3D human liver microtissue model using nonparenchymal cells and primary hepatocytes. The group followed a regimen of prolonged, repeated very low exposure concentrations of titanium dioxide (TiO_2_), cerium(iv) oxide (CeO_2_), and zinc oxide (ZnO) NPs. Then, the researchers allowed a two-week recovery period to mimic the liver's recovery capacity *in vivo*. Sub-lethal concentrations resulted in very low material-induced cell death, even after ten repeated exposures over 21 days. The study also reported a significant reduction in secreted albumin. Albumin is synthesized almost exclusively by hepatocytes, which is why its secretion rate is widely used *in vitro* as an index of retained hepatocyte synthetic function;^159^ it is not, however, a specific indicator of injury, and comparable information can be obtained from other hepatocyte-derived outputs such as fibrinogen, transferrin, or urea. In addition, the results show that oxidative stress and pro-inflammatory cytokine release increase in a time- and dose-dependent manner upon exposure to the NPs. Darkfield imaging showed that the NPs could penetrate the core of the spheroid, supporting the use of 3D models for investigating NP exposure and induced toxicity. However, cytotoxicity results showed that untreated control cell viability declined after the second week, suggesting that future long-term NP exposure studies should be limited to two weeks. In conclusion, the study showed that cumulative functional impairment became apparent after multiple exposures to several NPs, highlighting this system's capacity to model chronic hepatotoxicity.^160^ Another study developed liver multicellular spheroid models in three different hydrogel environments and used them to assess the cytotoxicity of AgNPs, ZnO NPs, and silicon dioxide (SiO_2_) NPs.^161^ The hydrogel was the variable under comparison: HepG2 cells were embedded in each of the three matrices so that spheroids formed within distinct extracellular environments, and the same panel of NPs was then applied to all of them, so that any difference in the measured cytotoxicity could be attributed to the surrounding matrix rather than to the particles themselves. The research group tested Matrigel, collagen type I, and gelatin as the ECM for the liver spheroid model to investigate how the microenvironment influences the toxic effects of the NPs. The study aimed to identify the optimal environment for cells in the 3D system and its impact on the outcomes of the cytotoxicity assessments. The results showed that HepG2 cells proliferated most when using Matrigel, likely because its high wettability and hydrophilicity are preferred by HepG2 cells. In addition, the results showed that 3D liver spheroid models showed lower NP-induced cytotoxicity as compared to 2D monolayer cells with the same exposure time and dose of NPs; the hydrogel and the cells in the outer layer of the liver spheroid protect the cells in the inner layer from NPs diffusion and penetration, hence lowering the NP-induced cytotoxicity. The study showed that 3D cell cultures better reflect *in vivo* conditions, improving the predictive outcome of NP-induced cytotoxicity.^161^

### 5.3. Kidney models

The kidney plays central physiological roles, including homeostasis, metabolism, and excretion of metabolic waste products and toxins.^162,163^ The functional unit of the kidney is the nephron, of which each human kidney contains approximately one million, comprising a glomerulus, a proximal tubule, the loop of Henle, a distal tubule, and a collecting duct. Blood entering the glomerulus is filtered across a trilaminar barrier formed by a fenestrated endothelium, the glomerular basement membrane, and the interdigitating foot processes of podocytes bridged by slit diaphragms, an arrangement that imposes both size and charge selectivity on everything leaving the circulation.^164^ This barrier restricts the passage of circulating macromolecules above roughly 60 kDa and, for engineered NPs, sets a filtration threshold near 5.5 nm hydrodynamic diameter, below which NPs are rapidly excreted in the urine and above which they are retained in circulation and repeatedly delivered to renal tissue.^165^ The filtrate then enters the proximal tubule, whose brush border epithelium reabsorbs most filtered solutes and proteins through the megalin–cubilin endocytic receptor complex, alongside apical and basolateral organic anion and cation transporters that actively concentrate xenobiotics within tubular cells.^166^ Because the kidneys receive approximately a quarter of the cardiac output while representing less than one percent of body mass, and because progressive water reabsorption concentrates the luminal contents along the nephron, the proximal tubule is exposed to substantially higher local concentrations of circulating compounds than most tissues and is consequently the principal site of nephrotoxic injury.^167^ A size- and charge-selective filtration barrier, receptor-mediated tubular reabsorption, transporter-driven intracellular accumulation, and a polarized, metabolically active epithelium are therefore the features that 3D kidney models are intended to reproduce.

*In vivo* studies consistently demonstrate that systemically bioavailable NPs can access the kidneys and undergo measurable biodistribution within renal tissues.^168^ This increases the need for safety evaluation of NPs and their metabolites. A significant concern in the development of NPs is nephrotoxicity, which refers to kidney dysfunction and damage upon exposure to high concentrations of toxic metabolites in the kidney's proximal tubules.^163^ Therefore, researchers have developed 3D kidney models that recapitulate *in vivo* tissue organization and physiological characteristics for nephrotoxicity assays.^169,170^ Researchers used unique culture conditions to preserve primary cells and develop a 3-D organoid kidney proximal tubule model. The study shows that the model has been validated for proximal tubule phenotype stability and cell functionality for 2 months.^169^ Another research group used this model to screen the toxicity of AuNPs and G5-OH PAMAM dendrimers, aiming to assess the 3D cell culture model's ability to replicate *in vivo* toxicity induced by these NPs. To assess the *in vitro* nephrotoxicity of both NPs, DNA and cell membrane integrity were evaluated, and immunohistochemistry for biomarkers and inflammatory cytokine assays were performed. The results demonstrated that the 3D kidney proximal tubule organoid model maintained key aspects of renal architecture and cell-matrix interactions and can effectively reproduce NP-induced nephrotoxicity with strong relevance to *in vivo* outcomes. The results highlighted that the G5-OH PAMAM dendrimers induced kidney injury biomarkers Kim-1 and TNFα, reflecting proximal tubule cellular damage. In addition, this 3D model improved prediction of *in vivo* toxicity compared with 2D cell cultures, as the observed biomarker responses and cell viability closely aligned with previously reported animal data. Finally, these findings support using 3D kidney organoids as a predictive *in vitro* platform for assessing NP-induced nephrotoxicity.^171^

### 5.4. Cancer models

Tumor spheroids are commonly used 3D cancer models for NP screening because they recreate many hallmark features of solid tumors while remaining relatively simple to generate and maintain.^37,66,67,172^ When cancer cells aggregate into compact multicellular spheroids, they naturally form gradients in oxygen, nutrients, and metabolites as diffusion becomes limited toward the center. This leads to the development of distinct spatial regions, an actively proliferating outer layer, a more quiescent intermediate zone, and often a necrotic core. This closely resembles the organization of poorly vascularized tumor tissue *in vivo*. For NP delivery studies, these structural features matter because they introduce the same biological and physical barriers that limit therapeutic penetration in patients. NPs must move through dense cell layers, overcome restricted diffusion in the inner regions, and interact with cells whose phenotypes have shifted under hypoxic and nutrient-limited conditions. As a result, NP uptake in spheroids is often slower than in 2D monolayers, and NPs accumulation is frequently concentrated near the spheroid periphery rather than evenly distributed throughout the structure. These outcomes offer a more realistic readout of whether a formulation can penetrate deeply enough to reach therapeutically relevant tumor compartments.^26^ A major advantage of 3D cancer models, as aforementioned, is their ability to incorporate key aspects of the tumor microenvironment (TME) that strongly influence NP delivery. ECM composition (*e.g.*, collagen, fibronectin, laminin), stromal populations (*e.g.*, fibroblasts, immune cells), and tumor-associated biophysical conditions (*e.g.*, hypoxia, altered cell mechanics) can collectively alter NP diffusion and cellular interactions. Dense ECM can act as a size- and charge-dependent barrier, limiting penetration of larger NPs and trapping cationic systems, while hypoxic and acidic conditions may impact NP stability and trigger premature or enhanced payload release in stimuli-responsive formulations. Co-culture spheroids further increase biological relevance by enabling assessment of NP biodistribution across multiple cell types and capturing immune-related effects absent in monoculture systems. However, spheroids typically lack perfusion, tissue interfaces, and dynamic mechanical cues that shape NP transport *in vivo*. To address these limitations, NAMs such as organ-on-a-chip systems incorporate microfluidic control to model perfusion, shear stress, and barrier function.^173,174^ These platforms enable NP testing under flow, providing more physiologically relevant readouts of particle margination, adhesion, and uptake. A widely used barrier model relevant to NP transport is the Caco-2 organ-on-a-chip system. Caco-2 cells form polarized epithelial monolayers with tight junctions that function as an intestinal barrier model, enabling assessment of nanocarrier permeability and transport across epithelial interfaces.^175^ While not a direct tumor model, this platform is highly relevant for cancer nanomedicine development because it enables mechanistic screening of NP translocation, barrier penetration, and absorption behavior, which are critical considerations for oral delivery strategies and for understanding epithelial barrier interactions in metastasis and tumor-associated tissues.^175^ Complementary tumor-relevant *in vitro* models are also widely used for early mechanistic screening of nanocarrier uptake and intracellular trafficking. For example, organoid breast cancer models such as MDA-MB-231 have been used to evaluate uptake of MSN formulations complexed with fluorescent NANPs, providing rapid readouts of internalization efficiency and subcellular distribution.^68^ Similarly, organoid pancreatic cancer cells (PANC-1) have been used to compare internalization profiles of PAMAM dendrimers (G3-G7) complexed with chemically distinct NANPs (DNA *vs.* RNA), enabling structure–activity assessment of how carrier generation and nucleic acid composition shape delivery outcomes.^66^ With this, microfluidic systems can be adapted to cancer contexts by integrating tumor spheroids/tumoroids/organoids, vascular endothelial channels, and stromal compartments in the same device, thereby enabling NP screening within a controlled TME under perfusion. These platforms support real-time imaging and high-content analysis, making them promising tools for more predictive NP selection prior to animal studies. These systems are also used for modeling the recruitment and infiltration of immune cells in tumors.^176,177^ Overall, 3D cancer models help close the gap between simplified 2D assays and the delivery barriers that dominate *in vivo* tumors. Tumor spheroids, tumoroids, and organoids are especially useful as scalable, accessible systems for measuring NP penetration and microenvironment-driven limits on uptake and efficacy. More advanced microfluidic and organ-on-a-chip platforms add another layer of realism by introducing perfusion, tissue–tissue interfaces, and transport under flow, features that strongly shape NP distribution and cellular interactions *in vivo*. Together, these models improve the translational value of NP screening by flagging penetration failures, microenvironment-dependent activity, and transport limitations early in development, ultimately supporting more rational nanocarrier design and better clinical predictability.

### 5.5. Placental models

The placental barrier has received special attention in nanomaterial toxicology because fetal development is a critical window during which exposure to nanomaterials may lead to detrimental health outcomes at birth and later in life. The placenta serves as a permeability barrier that regulates the exchange of nutrients, oxygen, waste products, and metabolites between the maternal and fetal circulations,^178^ while also functioning as a highly selective biological barrier that safeguards the developing fetus from harmful xenobiotics.

The cellular barrier separating maternal and fetal blood comprises the syncytiotrophoblast layer, which interfaces with the maternal environment, and the endothelial cell layer of the fetal microcapillaries.^179^ Between these two layers lie stromal cells such as cytotrophoblasts, fibroblasts, and Hofbauer cells, which resemble tissue macrophages; the cytotrophoblast layer itself partially disappears as gestation advances.^180^ Transfer of substances into fetal circulation is limited by a combination of mechanisms, including simple diffusion, transporter-mediated exchange, metabolic enzymes, and phagocytosis or pinocytosis. As exposure to NPs from environmental sources, vaccines, and therapeutics increases, concern has grown about their effects during pregnancy and early childhood.^181^ Rodent models provide evidence that NPs can cross the placental barrier; however, differences in human placental anatomy limit their predictive value for human outcomes.

NP toxicity and placental transfer are commonly evaluated using 2D models such as BeWo, JEG-3, or primary human trophoblast cultures, which offer a straightforward setup for assessing cellular uptake and initial cytotoxicity.

To better capture the structural complexity and fluid dynamics of the placental interface, researchers have developed more advanced 3D models, including microfluidic placenta-on-a-chip platforms, multicellular spheroids, and stem cell-derived organoids that simulate multi-layered tissue architecture and blood-flow shear stress. A study addressed the shortage of predictive platforms for NP-placenta interactions by developing a scaffold-free 3D co-culture microtissue model using hanging drop technology. The group assembled a core of placental fibroblasts enveloped by a trophoblast cell layer, reproducing the cellular arrangement of the villous tissue. Secretion of human chorionic gonadotropin was significantly higher in the 3D microtissues than in corresponding 2D cultures, indicating enhanced trophoblast differentiation, and the system proved reproducible enough to support acute toxicity testing of gold, titanium dioxide, cadmium telluride, and CuO NPs.^182^ The same platform was later used to determine how particle properties govern penetration into placental tissue. The group exposed microtissues to AuNPs differing in size (3–4 nm *vs.* 13–14 nm) and surface chemistry (sodium carboxylate *vs.* polyethylene glycol), quantified uptake by inductively coupled plasma mass spectrometry, and localized the particles by TEM and laser ablation inductively coupled plasma mass spectrometry. Smaller and carboxylate-modified NPs were taken up to a greater extent and penetrated deeper into the microtissue core, whereas larger or PEGylated NPs barely passed the outer trophoblast layer. In conclusion, the study demonstrated that placental uptake and penetration can be steered by tailoring particle size and surface modification, a prerequisite for designing carriers that either target or spare the placenta.^183^

A recent study used a dynamic placenta-on-chip model incorporating a modified placental microenvironment to examine liposomal uptake under physiologically relevant flow conditions. The group cultured trophoblasts under continuous perfusion and induced graded degrees of syncytialization with forskolin, characterizing the resulting phenotype quantitatively by beta human chorionic gonadotropin secretion and qualitatively by immunostaining of the tight junction protein ZO-1 and of F-actin to visualize microvilli formation. Uptake of chondroitin sulfate A-conjugated and control liposomes was then measured by confocal microscopy. Flow alone intrinsically induced syncytialization and microvilli formation relative to static controls, and uptake increased further with forskolin treatment, indicating a combined effect of shear stress and differentiation state on NP uptake.^184^ Another study extended the chip format to a complete barrier configuration to probe environmental NP exposure. The device recreated a near-physiological 3D microenvironment by combining extracellular matrix with flow in the maternal and fetal compartments and was challenged with TiO_2_ NPs. Oxidative stress, cell apoptosis, barrier permeability, and maternal immune cell behavior were assessed in parallel. In contrast to oxidative stress and apoptosis, barrier integrity and maternal immune cells were considerably affected even at low NP concentrations, suggesting that functional barrier endpoints are more sensitive than conventional cytotoxicity readouts.^185^ A further study replaced immortalized cell lines with human trophoblast stem cells to improve physiological fidelity. The stem cells were co-cultured with endothelial cells in a dynamic microsystem, where continuous fluid flow drove progressive differentiation into syncytiotrophoblast and the formation of a bilayered trophoblastic epithelium mimicking the structure of placental villi. Copper oxide (CuO) NPs introduced on the trophoblastic side to simulate maternal blood exposure hindered differentiation, diminishing hormone secretion and impairing glucose transport, disrupting autophagic flux, and inducing apoptosis. The model additionally captured the downstream inflammatory cascade, including maternal macrophage activation, inflammatory cytokine secretion, and endothelial barrier disruption.^186^

The next level above 3D models is placental explants (small fragments of dissected placental tissue), which retain the native villous architecture, to examine indirect effects mediated by the placental secretome. First-trimester and term explants were exposed to TiO_2_ and SiO_2_ NPs. Conditioned medium from the exposed explants altered endothelial spheroid sprouting, revealing material- and pregnancy-stage-specific dysregulation of secreted signaling factors with consequences for angiogenesis and vascularization even where NP transfer to the fetal circulation is limited.^187^

*Ex vivo* human placental perfusion of freshly delivered cotyledons remains the gold standard for directly measuring real-time transplacental transport and tissue-level effects in an intact human organ, offering a level of physiological relevance that *in vitro* models cannot fully replicate. An *ex vivo* placental perfusion method was used to measure transplacental transport of multiple NP platforms, such as AgNPs,^188^ polystyrene NPs,^189^ and LNPs.^190^

## 6. Cross-cutting observations for existing organ models

Read together, these organ-specific studies point to a small set of determinants that recur across tissues, but to thresholds that are organ-specific because each is imposed by a different anatomical constraint.

Hydrodynamic size and surface chemistry govern access in every system examined: gold NPs of 2 nm crossed the brain spheroid barrier whereas larger constructs did not;^152^ in placental microtissues, 3 to 4 nm carboxylate-modified particles penetrated the tissue core while 13 to 14 nm or PEGylated particles were retained in the outer trophoblast layer;^183^ renal clearance falls away above approximately 5.5 nm;^165^ and access to hepatic parenchyma is bounded by the diameter of the sinusoidal fenestrations. The governing variables are thus the same throughout, yet the numerical cut-off differs by an order of magnitude between organs, because it is set in one case by a tight junctional seal, in another by a multilayered trophoblast, in another by the glomerular slit diaphragm, and in another by an endothelial pore. A practical consequence follows: a carrier optimized to cross one barrier is not neutral with respect to the others, and size tuning intended to improve delivery to one organ predictably alters exposure elsewhere.

A second generalization is that dimensionality alone accounts for less of the difference between models than the mechanical and differentiation state of the cells within them. Perfusion of trophoblast cultures induced syncytialization and microvillus formation without any chemical stimulus and altered liposome uptake accordingly,^184^ and comparisons of identical titanium dioxide particles under static and dynamic conditions diverge for the same reason. Thus, 3D is not a single experimental variable, and penetration data are not comparable between laboratories unless shear stress and differentiation markers are reported alongside particle characteristics. This is a reporting deficit rather than a biological obstacle, and it is therefore tractable.

A third, and less comfortable, observation is that the same finding is currently offered both as evidence of model fidelity and as evidence of model limitation. Reduced cytotoxicity in 3D multi-layered relative to monolayer culture is attributed to protection of inner cells by the surrounding matrix and outer cell layers,^161^ but an outer layer that takes up particles is simultaneously a barrier and a sink, and diffusion limitation yields the same result as genuine cytoprotection. Separating the two requires the dose delivered to the cells to be measured rather than inferred from the concentration applied. Until delivered dose is reported, the reduced sensitivity of 3D systems cannot be interpreted, and the observation supports neither the claim that such models are more predictive nor the claim that they are less sensitive. However, this limitation only applies when there are multiple cell layers, and 3D cell culture models comprised of monolayers do not face this obstacle.

Finally, a consistent ordering of endpoint sensitivity emerges across otherwise unrelated systems. In the placental chip, barrier integrity and maternal immune cell responses were affected at concentrations that left oxidative stress and apoptosis unchanged;^185^ in placental explants, the secretome was dysregulated with consequences for angiogenesis in the absence of detectable cytotoxicity;^187^ and in liver microtissues, albumin secretion declined while cell death remained minimal.^160^ Functional, secretory and barrier endpoints therefore respond before viability does, which implies that screening cascades built upon viability assays will systematically underestimate hazard, and that the value added by 3D systems lies less in their architecture than in the functional readouts that architecture makes available. What the literature does not yet contain is a study in which one particle is examined across monolayer, spheroid, chip and perfused tissue with delivered dose held constant; until such comparisons exist, the relative fidelity of these formats cannot be ranked.

## 7. Regulatory agencies’ perspectives

Based on the advances in science that collectively improved understanding of nanotechnologies, regulatory agencies have developed and published numerous guidance documents to help researchers with translating nanotechnology-based concepts from bench to clinic. These recommendations are available in detail elsewhere^191–197^ and are not covered in this review, which focuses on NAMs. Over the past two decades, NAMs have been greatly integrated into nonclinical drug development programs.^20^ This is supported by a recent report analyzing the use of NAMs over the past 15 years in investigational new drug (IND) applications, new drug applications (NDAs), and biologics license applications (BLAs) submitted to the U.S. FDA CDER, showcasing that scientists have already started implementing NAMs in their experimental research to reduce animal testing.^198^ The U.S. FDA CDER report disclosed that the most common NAMs applied fall under the *in silico* (49% of total submissions) and *in vitro* categories (44% of total submissions). When *in vitro* assays were used, these submissions included 3D models and organ-on-a-chip or microphysiological systems (MPS) models.^198^

U.S. FDA CDER has also shared its views on both the utility of using NAMs in the context of the weight of evidence approach and its limitations for the purposes of the drug approval process.^199–202^ More recently, U.S. FDA CDER also shared the toxicology reviewer's perspective with detailed case studies and review assessment examples, including NAMs use for identification of biomarkers for a rare disease, understanding mechanisms of toxicity, investigating species sensitivity to gastrointestinal toxicity, and assessing the risk of drug-induced liver injury.^20^

U.S. FDA Center for Biologics Evaluation and Research (CBER) disclosed that since 2016, U.S. FDA CBER received 70 investigational new drug applications for vaccines, gene and cell therapies, utilizing artificial intelligence (AI) and machine learning (ML) algorithms to predict, cluster, classify, and detect anomalies in these products.^203^ By organizing several public workshops, U.S. FDA CBER collected information on the use of AI and ML approaches in the development of biologics and published a white paper describing the U.S. FDA CBER's current position on AI/ML and requesting public feedback to develop guidance for industry.^204^

In its 2025 roadmap document, the U.S. FDA encouraged drug developers to use NAMs; however, the roadmap for the first three years focuses on monoclonal antibodies, which have been in use for decades and for which a wealth of preclinical and clinical safety data is already available.^205^ As science advances for other types of biologics and drugs, the NAM framework is expected to extend to these products as well. The areas of drug development actively exploring these methodologies include physiologically based pharmacokinetic (PBPK), immunogenicity, and toxicology. According to this roadmap, 17 federal agencies, including the U.S. FDA and NIH, will coordinate their NAMs standardization, education, and data sharing community outreach efforts through the existing interagency coordinating committee on the validation of alternative methods (ICCVAM).^205^

Similar efforts are undertaken by the EMA, which shared their experiences, perspectives, challenges, and recommendations for using NAMs in drug development through the 2025 EU-IN Horizon scanning report.^206^ While the agency has encouraged using NAMs for decades, the use of NAMs in regulatory submissions to the EMA is currently limited.^207^ Small and medium enterprises and federally funded academic institutions in the European Union lead the development of NAMs methodologies and focus on their implementation into the drug development process, as judged by requests for information they submitted to the EMA between 2019 and 2023, with popular topics including drug safety and toxicology, pharmacokinetics and biodistribution, developing liver and brain models, novel 2D models, and organs on a chip.^207^ In contrast, the majority of big pharma's requests regarding NAMs focus on using the data generated using these models in lieu of currently required animal testing, with popular areas concerning biosimilar comparability and immunogenicity.^207^ The EMA uses these interactions to reach consensus and implement a weight- of-evidence justification for omitting animal safety and toxicology studies.^207^

## 8. Limitations and challenges of *in vitro* NAMs

Despite NPs being evaluated in organoid and tumoroid models in comparison to conventional 2D cell cultures and animal models, the main limitation of these studies is that the comparisons were not conducted in a systematic way, with most data either NP platform- (*e.g.*, liposomes, metal, or polymeric NPs) or organ- (*e.g.*, brain, breast, or pancreas) specific.^208–210^ Moreover, the comparisons were made using research-grade nanomaterials, representing a platform or a prototype rather than marketed nanomedicine (*e.g.*, PEGylated liposomal doxorubicin (Doxil), nanoalbumin-formulated paclitaxel (Abraxane), PEGylated liposomal irinotecan (Onivyde)) and did not include a comparison to clinical safety and data used to gain marketing approval for these nanomedicines. The value of the existing studies using prototypes, platforms, and preclinical models is that they allowed identifying some limitations of 3D organoids in the context of NP physicochemical properties. For example, organoid penetration is limited for NPs with sizes greater than 100 nm, whereas data on the role of surface charge are controversial (reviewed in ref. 211), suggesting that many factors, including the type of nanocarrier (*e.g.*, LNPs *vs.* AuNPs), synthesis procedures, potential impurities and inconsistencies, and related physicochemical properties (*e.g.*, shape, rigidity, and surface functionalization) all contribute to the study outcome.

Disease-specific limitations were also identified. For example, in tumor organoids, the predictive value is high for epithelial solid tumors, in which tissues are more accessible, the tumor is genetically driven, and the clinical outcome depends on the intrinsic tumor sensitivity to a drug.^208^ Organoid models are less predictive when tumor targeting and therapeutic outcomes depend heavily on complex physiological processes (*e.g.*, angiogenesis and organ-specific physiology, including the overall hormonal balance and neuroendocrine interactions), interactions within the tumor stroma, and systemic immunity.^212–214^ Modeling tumors in which efficacy depends on drug pharmacokinetics, which is particularly relevant to NP-formulated drugs, especially those formulations with conditional drug release (*e.g.*, pH-, ultrasound-, or temperature-driven), as reviewed in detail elsewhere,^215–218^ poses significant challenges for current tumor organoids because there is often no straightforward approach to vascularization, establishing physiologically relevant fluid dynamics, and applying intended drug-release conditions in the organoid model.

While two approaches are currently used to achieve immune cell presence in tumoroid models, immune cells are either expanded during organoid formation from iPSCs or immune cells from peripheral blood are added to organoid co-culture,^212^ they do not recapitulate the breadth of the immune cell phenotypes present in the tumor and have limited viability over the culture time (discussed in ref. 212). This has led some experts to warn the organoid research community that organoid models can barely recapitulate the breadth of immune cell phenotypes and their functionality in the tumor microenvironment.^212^ One potential solution to this limitation under investigation is the air–liquid interface (ALI) technique, which includes encapsulation of organoids in a collagen gel, with one side exposed to air and the other to the culture medium, to retain endogenous stromal and immune cells. For colorectal and lung cancer organoids, this approach retained leukocytes for 10 days, although maintaining the T-cell population beyond this time was challenging.^213^ A modified ALI technique was more successful in both the breadth and longevity of immune-infiltrating organoid culture: it produced a broader immune cell population (NK cells, T- and B-lymphocytes, and macrophages) and longer culture times (over several months).^214^

Establishing vascularized organoids is also challenging. Current approaches include either implantation of the initial organoid into highly vascularized tissues in live animals to allow host vasculature to infiltrate the organoid, followed by extracting this *in vivo* vascularized organoid from the animal^219^ or co-culturing mixed cells using gene-editing and compartmentalized microfluidic chips to create a vascular endothelial growth factor (VEGF) and hypoxia gradient for guiding vascularization.^219,220^ High tenacity and costs of creating vascularized organoid models, the requirement for highly skilled personnel performing animal surgery and gene editing, and the need for supporting infrastructure create entry barriers for establishing these models and challenges with the model's reproducibility. Microphysiological systems offer an alternative approach that can provide perfusable vasculature under fluid flow, either *via* pumps or gravity-driven perfusion.^221^

General logistical challenges include the breadth of protocols, sources of starting tissues, iPSCs, and other essential materials and supplies, along with culture conditions, even for the same type of organoid model (reviewed in ref. 23, 222 and 223), as well as specialized training, which collectively make the reproducibility of models and data generated using these models challenging. While mimicking the heterogeneity of organs is an advantageous property, the heterogeneity of cells in organoids represents yet another logistical and technical challenge. For long-term cultures, periodic characterization and tracking of genotypes and phenotypes are required to ensure consistency across experiments, as less abundant, slow-growing cells can be lost and replaced by dominant, fast-growing cells over time, shifting the model's characteristics and compromising predictive value.^209^ The development and offering of a growing number of off-the-shelf cell products from NAM developers is a current approach to standardize models and overcome lab-to-lab variation.

Regulatory challenges include the lack of benchmarks, reference standards, generally agreed-upon standard practices, terminology, acceptance criteria for performance qualification and validation of organoid models, and regulatory guidance for industry. As more regulators disclose their experience with NAMs’ use in the drug approval process, such as recent reviews by the U.S. FDA CDER,^20,199,200^ the current regulatory gaps are expected to narrow, equipping the research community with tools to optimize and expand the use of organoid models in preclinical studies.

## 9. Computational NAMs

### 9.1. *In silico* NAMs for NP assessment

Regulatory roadmaps increasingly include computational modeling as part of the NAMs toolbox, together with advanced *in vitro* systems, to support evidence generation while reducing reliance on animal studies.^20,205,224^ For NPs, the main reason to use computational NAMs is the mismatch between experimental throughput and the complexity of the formulation design space. Composition, size, dispersity, surface chemistry, ligand density, and stability in biologically relevant media can vary independently, while measured responses can shift with dispersion preparation, dosing format, exposure time, and medium composition.^225^ In this context, *in silico* NAMs are best viewed as decision-support tools. They can prioritize formulations, interpret exposure-dependent responses, and generate mechanistic hypotheses, if model inputs are specified rigorously, dose is defined reproducibly, and the limits of use are stated explicitly. For NP assessment, these approaches include data-driven prediction for candidate ranking, mechanistic modeling for transport and exposure, PBPK/ODE (ordinary differential equations) models for dynamic disposition and kinetics, and molecular or multiscale modeling for NP-biological interface processes that alter particle identity (Fig. 5).

**Fig. 5:**
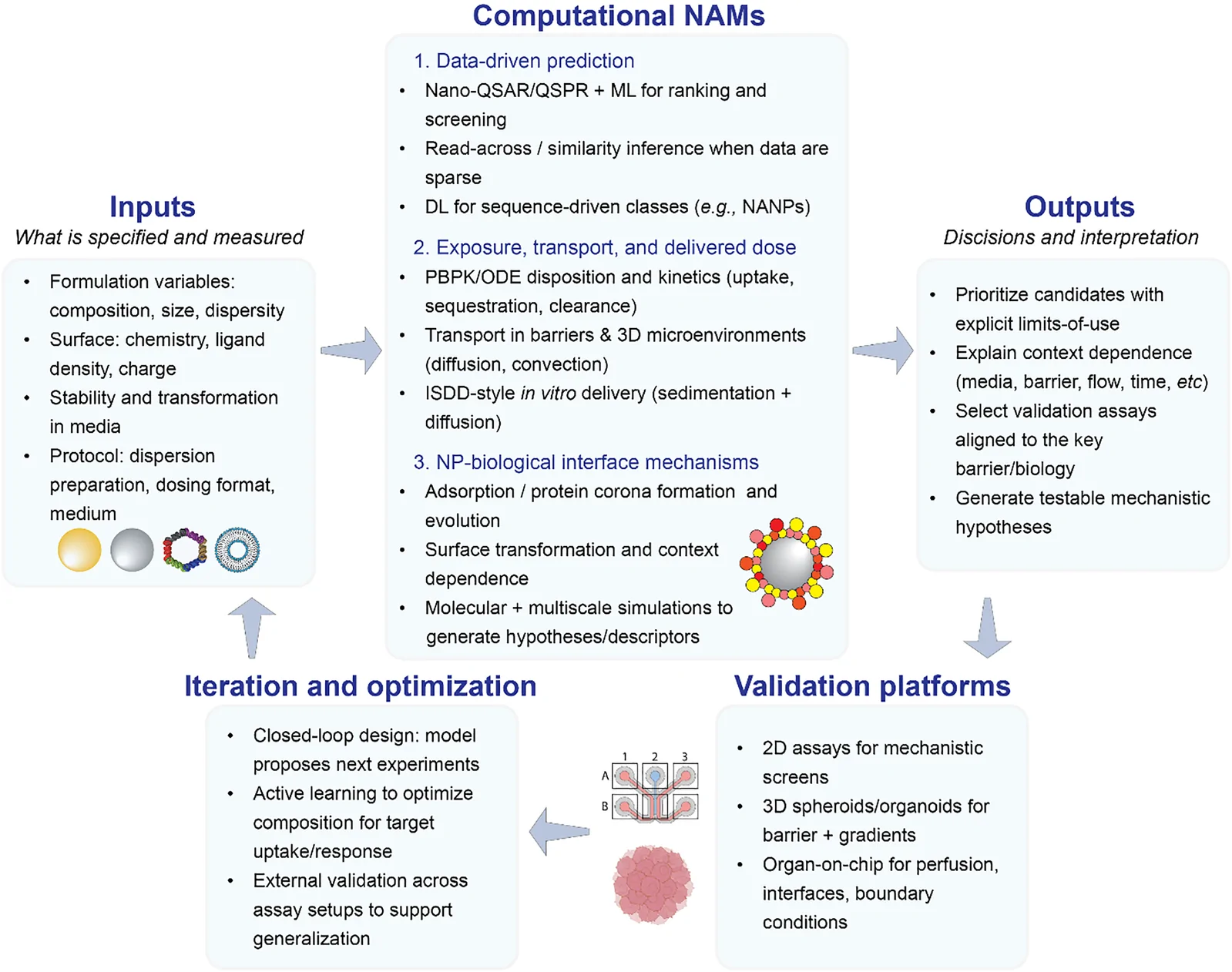
Computational NAM workflow for NP assessment. The workflow links NP inputs and assay context to three complementary classes of *in silico* models: (1) data-driven prediction (nano-QSAR/QSPR, machine learning, read-across, and deep learning), (2) exposure, transport, and delivered-dose modeling (including PBPK/ODE frameworks, transport in barriers and 3D systems, and ISDD-style dosimetry), and (3) NP-biological interface modeling (including adsorption, protein corona formation, surface transformation, and molecular/multiscale simulations). Together, these approaches support candidate prioritization, exposure-aware interpretation, mechanistic hypothesis generation, and selection of validation assays. Model outputs are evaluated experimentally using 2D assays, 3D spheroids/organoids, and organ-on-chip platforms, with iterative feedback used to refine both experiments and models.

### 9.2. Systems-level modeling: PBPK and ODE frameworks

Computational NAMs are most useful when they do more than predict an endpoint. For NP assessment, their central value is to connect formulation properties to exposure, transport, tissue distribution, and biological response across scales. PBPK modeling provides one established systems-level framework for this task by representing organs and tissues as connected compartments and estimating tissue partitioning, clearance, and systemic exposure over time.^226,227^ In nanomedicine, this framing matters because NP behavior often depends on processes not captured by nominal concentration alone, including vascular transport, opsonization, mononuclear phagocyte system uptake, tissue retention, renal or hepatobiliary clearance, and condition-dependent cargo release.^226,227^ ODE-based models provide a complementary language for describing dynamic processes such as particle deposition, cellular uptake, endosomal escape, degradation, and payload release over time.^228,229^ Together, PBPK and ODE approaches define the kinetic and exposure context in which downstream nano-QSAR/ML models, transport simulations, and molecular-scale models should be interpreted. They also connect naturally to 3D and organ-on-chip platforms, where delivered dose, residence time, barrier transport, and tissue-specific responses can be measured more directly than in conventional static assays.

### 9.3. Data-driven prediction: nano-QSAR/QSPR and machine learning

Nano quantitative structure–activity/property relationships (nano-QSAR/QSPR) and machine learning models connect NP descriptors to outcomes such as uptake, cytotoxicity, barrier disruption, and immune activation.^230^ Their performance depends strongly on whether the experimental metadata are sufficiently complete and whether the selected descriptors represent the material state that was actually tested. This is a central issue for NPs, where dispersion protocol, medium composition, protein content, dosing format, and characterization conditions can shift both exposure and biological identity. MIRIBEL was proposed as a reporting standard for bio-nano experimental studies, defining the minimum information needed across material characterization, biological characterization, and experimental protocols to improve reproducibility and allow for quantitative comparisons and *in silico* modeling.^117^ For protein corona studies, MINBE provides a complementary checklist for bio-corona experiments, improving comparability and reuse of data generated across nanoscience, proteomics, and bioinformatics workflows.^231^ At the infrastructure level, eNanoMapper illustrates what is needed for reproducible modeling by combining a nanosafety database, standardized terminology, and programmatic access to nanomaterial descriptors and assay readouts suitable for nano-QSAR analyses.^232^

Representation-first learning is particularly useful for NP classes where the natural input is not a fixed descriptor table. NANPs provide a clear proof of concept because sequence, architecture, and carrier conditions can be treated as design variables. Within the AI-cell framework, a transformer-based model was trained on systematically characterized datasets of NANP-mediated immune responses from human PBMC assays to predict specific cytokine-associated responses from user-submitted NANP designs.^44,233–236^ The model-development workflow includes partitioning the dataset into training and external test sets, transformer-based model training, cross-validation, and independent validation before final model deployment (Fig. 6). This is a NAM-relevant example because it links chemically defined nanoscale architecture to a human-relevant immunological endpoint and enables rapid *in silico* prioritization before experimental testing. The same sequence-to-response logic was extended to a neuroimmune setting, where immunological responses were predicted in human microglia.^43^ More broadly, this example illustrates how native representations can be used when NP design variables are structured: sequences for NANPs, graph-like descriptions for ligand-decorated surfaces, or formulation embeddings for multicomponent carriers.

**Fig. 6:**
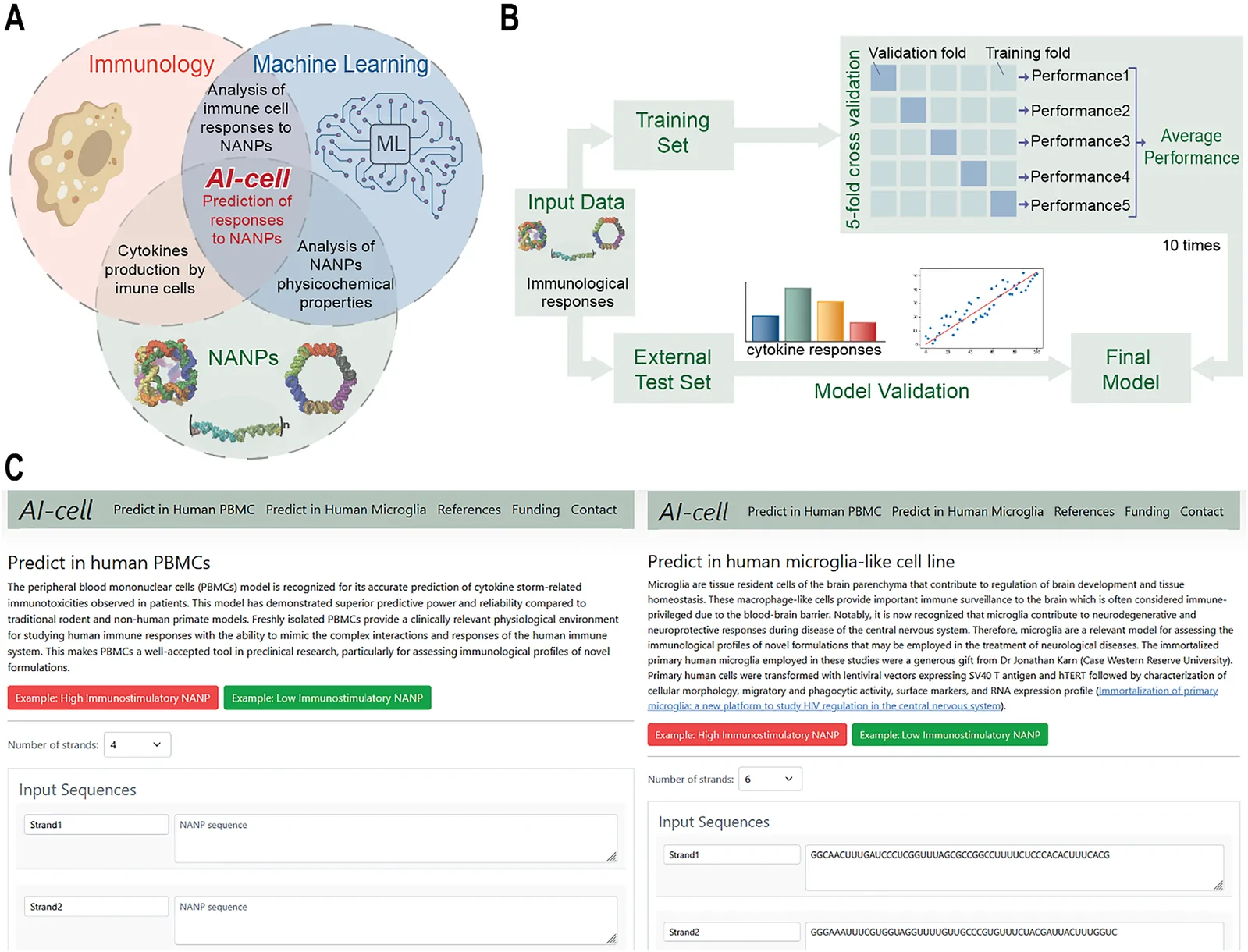
AI-cell as a proof-of-concept computational NAM for predicting immune responses to NANPs. (A) Conceptual overview of AI-cell, which integrates NANP design, immunological profiling, and machine learning to predict immune responses to NANPs. (B) Model development and validation workflow showing NANP dataset partitioning, transformer-based learning, cross-validation, external test-set evaluation, and final model generation (conceptual representation adapted from Small 2025, 21, e09459). (C) Web-based implementation of AI-cell, where user-submitted NANP sequences or designs can be screened for predicted cytokine/interferon-associated immune responses to support prioritization before experimental PBMC or microglia testing (https://aicell.ncats.io/).

A complementary example comes from active-learning-guided NP formulation, where microfluidic synthesis, high-content imaging, and iterative ML were combined to optimize PLGA-PEG NP composition for cellular uptake across two model-guided cycles.^237^ When training data are limited, sparse across formulations, or heterogeneous across assays, similarity-based inference remains useful. Nano-lazar adapts a read-across framework to NP toxicities using descriptors derived from core and coating structures, providing a practical option for data-limited toxicological prioritization.^238^ Together, these examples show that AI/ML integration in nanomedicine is most convincing when it is tied to a concrete decision: predicting an immune response, ranking a formulation, selecting the next experiment, or determining whether a new NP lies within the model's applicability domain.

### 9.4. Mechanistic modeling of exposure, transport, and dose

Response prediction alone is rarely sufficient for NP assessment because nominal concentration often does not reflect the exposure experienced by cells or tissues. Building on the systems-level PBPK/ODE framing above, mechanistic exposure models translate administered dose into biologically relevant metrics such as delivered dose, tissue residence time, spatial distribution, sequestration, clearance, and condition-dependent transformation. This is especially important for NPs, where transport barriers, particle deposition, agglomeration, dissolution, and medium-dependent changes can alter observed responses even when intrinsic cell-level interactions are unchanged.^226,227^ Transport modeling complements PBPK when spatial delivery restrictions matter, such as diffusion and convection in tissues and penetration into structured microenvironments. A computational model for diffusive NP transport in 3D tumor spheroids was developed and validated, linking measurable parameters to time- and concentration-dependent penetration behavior.^239^ Reviews about tumor transport also highlight the importance of microenvironmental barriers and binding interactions in controlling penetration, even when systemic exposure stays the same.^240^ Delivered dose is a key issue in both data-driven and mechanistic models. The *in vitro* sedimentation, diffusion and dosimetry (ISDD) model outlined how sedimentation and diffusion affect delivered dose *in vitro* and showed agreement with measured transport rates and cellular doses across various particle types.^241^

### 9.5. Molecular and multiscale modeling at the NP-biological interface

At the molecular scale, the central question is not only what an NP is at synthesis, but what biological surface it presents after entering a specific medium or tissue environment. Protein adsorption, corona formation, lipid binding, ligand shielding, dissolution, and surface restructuring can all change effective size, surface charge, receptor engagement, membrane association, opsonization, uptake route, and immune recognition. Molecular and multiscale simulations are most useful when they convert these processes into testable mechanistic hypotheses rather than broad qualitative explanations. For example, all-atom, coarse-grained, and mesoscale simulations can be used to ask which proteins preferentially adsorb to a given surface, whether adsorption masks a targeting ligand, how corona composition changes particle–membrane interactions, or whether a formulation is likely to favor membrane wrapping and internalization.^242,243^ These outputs can then be translated into experimentally measurable descriptors, such as adsorption propensity, corona stability, ligand accessibility, predicted membrane affinity, aggregation tendency, or surface transformation state. Such descriptors can be tested using corona proteomics, hydrodynamic size and zeta-potential measurements, uptake assays, membrane-interaction studies, or spatial readouts in 3D and organ-on-chip models. The main value of molecular and multiscale modeling is therefore not standalone prediction of complex *in vivo* efficacy or toxicity. Its value is to explain why NP responses shift across media, protein environments, and assay formats, and to generate mechanistically grounded variables that can improve higher-level ML, transport, PBPK, and ODE-based models.

## 10. Limitations and challenges of *in silico* NAMs

*In silico* NAMs for NP assessment are powerful, but their limitations are structural rather than incremental. The first constraint is data: most nano-QSAR/ML models are trained on heterogeneous studies, where key factors like dispersion protocol, media composition, protein content, dosing format, time, and characterization conditions are often inconsistently reported. As a result, the model often learns laboratory-specific artifacts rather than transferable nano-biological relationships.^117^ A second constraint is that the nominal dose does not effectively represent exposure in NP systems. Sedimentation, diffusion, agglomeration, and time-dependent changes separate the administered concentration from the dose delivered at the cell interface. The ISDD framework highlights this problem and reminds us that many model failures are actually dosimetry failures.^241^ Additionally, the identity of NPs depends on the context. The formation of a protein corona and surface changes can alter the effective size, charge, targeting, and immunorecognition of NPs. This makes predictions unreliable when transferring between different media, protein environments, or assay formats unless those conditions are clearly documented.^244^ Third, generalization is hard to justify without an explicit applicability domain and uncertainty reporting. Recent analyses show that even when an applicability domain is defined, it does not always align cleanly with prediction reliability, underscoring the need for uncertainty-aware use and prospective testing rather than relying on internal validation alone.^244^ Finally, using these models for regulatory purposes demands transparency, a defined context of use, and justifiable uncertainty. The organization for economic co-operation and development (OECD) guidance for (Q)SAR assessments emphasizes defined domains, reproducibility, and a structured evaluation of prediction reliability. These principles are even more critical for NPs, where exposure and transformation cannot be separated from “structure”.^245^ In practice, these limitations highlight the need for integrated workflows. Models should be used within well-defined contexts, grounded in exposure-aware measurements, and updated as standardized, model-ready datasets grow. This is also why integrating standardized 3D and organ-on-chip assays is essential. They provide the exposure-aware data needed to validate, calibrate, and refine models.

## 11. Future directions & conclusion

3D culture models and organ-on-chip platforms are now widely used in disease modeling and preclinical drug discovery because they recover key features missing from 2D assays, including multicellular architecture, gradients, and tissue-specific function.^246,247^ Organ-on-chip platforms extend this by adding controlled perfusion, mechanical cues, and tissue–tissue interfaces and have been used to reproduce clinically relevant drug responses and human pathophysiology *in vitro*.^248^ For NPs, these same features are often crucial because transport, localization, and delivered dose rely heavily on 3D barriers and flow-related exposure. This makes 3D/organ-on-chip readouts a fitting basis for exposure-focused modeling and design improvement.^249^*In silico* NAMs are most useful when combinatorially paired with 3D models that produce exposure-aware, tissue-relevant readouts in a consistent format. They also work well with organ-on-chip systems, where perfusion and barrier transport are key to understanding the biology.^248^ This connection is important because many results depend more on transport than on interactions. Static 3D systems capture the limits of the microenvironment, but they often fail to represent changing exposure conditions that affect how materials deposit and distribute. Organ-on-chip systems can clearly define these boundary conditions and make them measurable. A representative example is the microengineered human blood–brain barrier platform, which enabled 3D mapping of NP distributions in vascular and perivascular regions, linking barrier function to spatial delivery outcomes rather than relying on bulk uptake proxies.^250^ Importantly, this integration is already being described as a relevant direction for NAMs in the NP community. A recent review of organ-on-chip platforms that focus on testing NP toxicity and effectiveness outlined the field using a practical set of trade-offs, including physiological accuracy *versus* scalability, and technical complexity *versus* the validation and regulatory approval needed for regular use.^251^ On the modeling side, *in silico* strategies for nano-drug design across molecular modeling, machine learning, computational fluid dynamics, and related approaches were surveyed, and it was reported that progress is ultimately constrained by data quality and the gap between prediction and experimental validation.^252^ Altogether, these perspectives highlight a specific workflow requirement for NAM-grade use: 3D and organ-on-chip assays should report delivered dose and key covariates in a format ready for modeling, so that both machine learning and mechanistic models generalize beyond a single assay setup.

As non-animal and microphysiological approaches become more widely used, the field must maintain a realistic view of the predictive value of preclinical models. Deploying *in silico*, *in vitro*, and *ex vivo* platforms is critical for upholding the 3Rs principles and resolving early mechanistic questions. However, no single model captures the full physiological complexity of systemic responses across the human body. Rather than treating NAMs and animal models as mutually exclusive, preclinical evaluation must evolve into a cohesive, cross-validated, complementary testing framework. Regardless of the platform (*in vitro*, *ex vivo*, *in silico*, or *in vivo*), preclinical models cannot provide absolute certainty about clinical safety or efficacy in humans. Instead, their true value lies in systematic risk mitigation and in building the mechanistic evidence base necessary for clinicians to responsibly advance new formulations into clinical trials.

Historically, 3D assays were too heterogeneous and low- throughput to support dataset sizes needed for reliable modeling. Automation is narrowing that gap. A scalable, high-throughput screening (HTS)-compatible workflow for automated generation, maintenance, and optical analysis of human midbrain organoids in standard 96-well plates illustrates how standardized handling and quantitative imaging can make complex 3D biology compatible with screening-style experimentation and model-ready outputs.^253^ Once 3D and organ-on-chip platforms can produce structured datasets, AI can shift from retrospective prediction to prospective design. An example of a closed-loop pipeline that integrates microfluidic NP formulation, high-content imaging, and active machine learning to optimize the PLGA-PEG NP composition for uptake by human breast cancer cells across two ML-guided iterations is demonstrated.^237^ The way forward is a coupled NAM stack: standardized 3D and organ-on-chip assays that report delivered dose and interoperable data structures that can support external validation and closed-loop optimization, in which computation proposes experiments and advanced *in vitro* systems provide exposure-aware feedback that 2D cannot^251^ (Fig. 7).

**Fig. 7:**
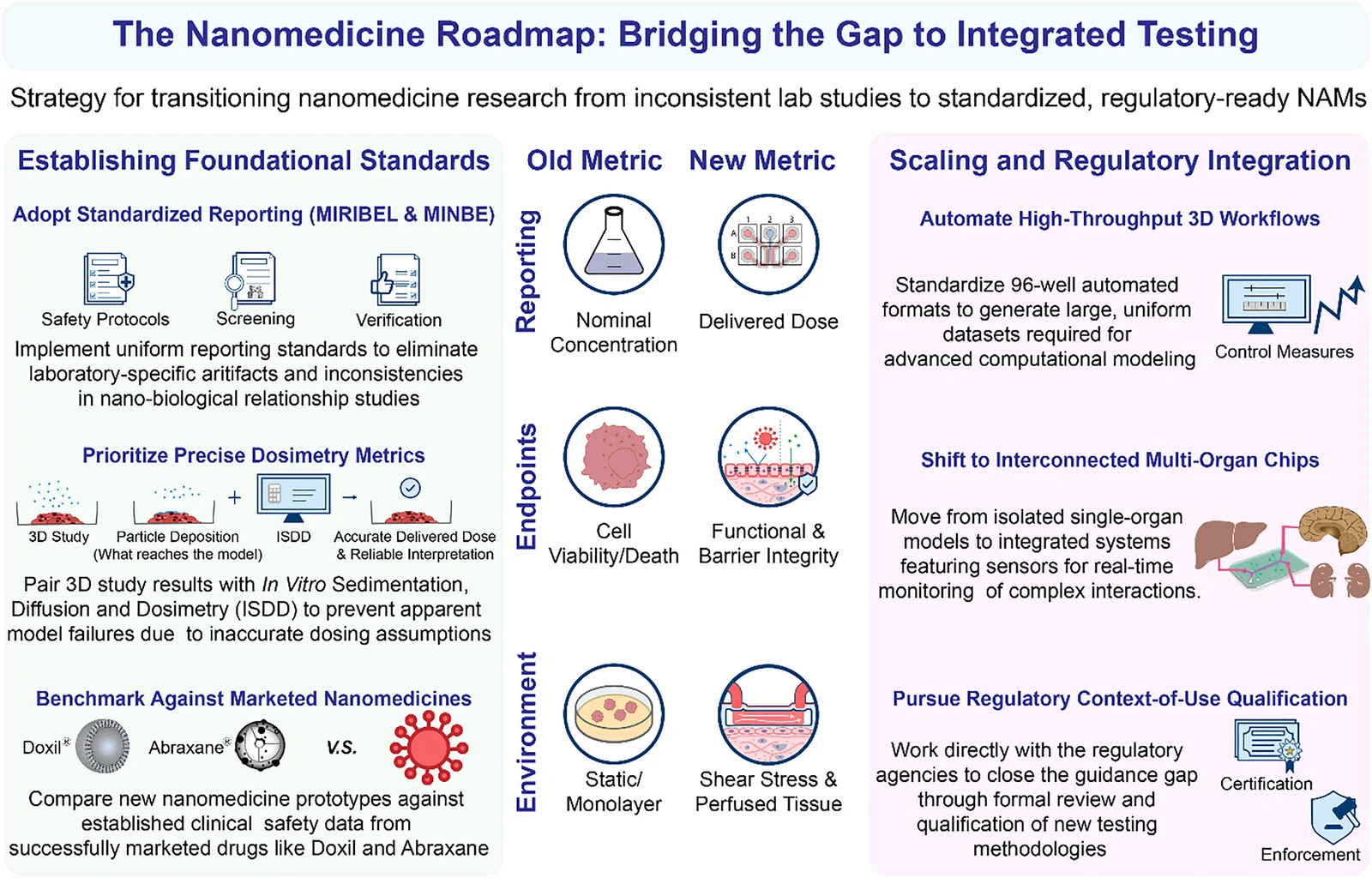
A roadmap for translating nanomedicine testing into standardized, regulatory-ready NAMs. Three foundational standards (left): adoption of uniform reporting frameworks (MIRIBEL, minimum information reporting in bio–nano experimental literature; MINBE, minimum information in nano-biology experiments) to eliminate laboratory-specific artifacts; pairing 3D culture data with the *in vitro* sedimentation, diffusion and dosimetry (ISDD) model to recover delivered dose and distinguish genuine model failure from inaccurate dosing assumptions; and benchmarking new prototypes against clinically established nanomedicines such as Doxil® and Abraxane®. The accompanying shift in measurement conventions (center) moves reporting from nominal concentration to delivered dose, shifts endpoints from viability to function and barrier integrity, and changes the culture environment from static monolayers to perfused tissue under shear. Scale-up and regulatory uptake (right) require automated 96-well 3D workflows that generate datasets large enough for computational modeling, progression from single-organ to sensor-equipped multi-organ chips, and direct engagement with agencies for context-of-use qualification.

In addition, to advance personalized medicine, multi-organ 3D models are being developed,^254^ in which the interactive nature of the human body is reflected by interconnected organoids engineered within microfluidic environments that enable inter-organ crosstalk.^255^ These models, along with sensor-integrated chips,^256^ enable real-time functional monitoring and enhance the predictive power of NPs' efficacy and toxicity.

In conclusion, all these efforts should be directed toward the translational potential of NAMs in the NPs' therapeutic landscape,^257^ and geared towards the 3R in animal testing, accelerating precision therapeutics, and enhancing clinical trial predictions. This goal will be achieved when interdisciplinary collaborative efforts of biologists, bioengineers, clinicians, and regulatory agencies are made to address related challenges, including validation, standardization, and scalability.

## Conflicts of interest

There are no conflicts to declare.

## Data Availability

As this is a review article, no new primary datasets were generated or analyzed in this study. All data and supporting information discussed in this article are available within the manuscript and through the cited references. Additional information may be obtained from the corresponding author upon reasonable request.
